# Temporal Lobe Epilepsy Perturbs the Brain‐Wide Excitation‐Inhibition Balance: Associations with Microcircuit Organization, Clinical Parameters, and Cognitive Dysfunction

**DOI:** 10.1002/advs.202406835

**Published:** 2025-01-13

**Authors:** Ke Xie, Jessica Royer, Raul Rodriguez‐Cruces, Linda Horwood, Alexander Ngo, Thaera Arafat, Hans Auer, Ella Sahlas, Judy Chen, Yigu Zhou, Sofie L. Valk, Seok‐Jun Hong, Birgit Frauscher, Raluca Pana, Andrea Bernasconi, Neda Bernasconi, Luis Concha, Boris C. Bernhardt

**Affiliations:** ^1^ McConnell Brain Imaging Centre Montreal Neurological Institute and Hospital McGill University Montreal QC H3A 2B4 Canada; ^2^ Otto Hahn Research Group for Cognitive Neurogenetics Max Planck Institute for Human Cognitive and Brain Sciences 04103 Leipzig Germany; ^3^ Institute of Neurosciences and Medicine (INM‐7) Research Centre Jülich 52428 Jülich Germany; ^4^ Institute of Systems Neuroscience Heinrich Heine University Düsseldorf 40225 Düsseldorf Germany; ^5^ Center for Neuroscience Imaging Research Institute for Basic Science Sungkyunkwan University Suwon 34126 South Korea; ^6^ Department of Biomedical Engineering Sungkyunkwan University Suwon 16419 South Korea; ^7^ Center for the Developing Brain Child Mind Institute New York City NY 10022 USA; ^8^ Department of Neurology and Department of Biomedical Engineering Duke University Durham NC 27704 USA; ^9^ Montreal Neurological Institute and Hospital McGill University Montreal QC H3A 2B4 Canada; ^10^ Institute of Neurobiology Universidad Nacional Autónoma de Mexico Queretaro 76230 Mexico

**Keywords:** cognitive impairment, excitation/inhibition imbalance, microcircuit perturbation, multimodal MRI, temporal lobe epilepsy

## Abstract

Excitation‐inhibition (E/I) imbalance is theorized as a key mechanism in the pathophysiology of epilepsy, with ample research focusing on elucidating its cellular manifestations. However, few studies investigate E/I imbalance at the macroscale, whole‐brain level, and its microcircuit‐level mechanisms and clinical significance remain incompletely understood. Here, the Hurst exponent, an index of the E/I ratio, is computed from resting‐state fMRI time series, and microcircuit parameters are simulated using biophysical models. A broad decrease in the Hurst exponent is observed in pharmaco‐resistant temporal lobe epilepsy (TLE), suggesting more excitable network dynamics. Connectome decoders point to temporolimbic and frontocentral cortices as plausible network epicenters of E/I imbalance. Furthermore, computational simulations reveal that enhancing cortical excitability in TLE reflects atypical increases in recurrent connection strength of local neuronal ensembles. Mixed cross‐sectional and longitudinal analyses show stronger E/I ratio elevation in patients with longer disease duration, more frequent electroclinical seizures as well as interictal epileptic spikes, and worse cognitive functioning. Hurst exponent‐informed classifiers discriminate patients from healthy controls with high accuracy (72.4% [57.5%–82.5%]). Replicated in an independent dataset, this work provides in vivo evidence of a macroscale shift in E/I balance in TLE patients and points to progressive functional imbalances that relate to cognitive decline.

## Graphical abstract

Xie and colleagues apply advanced signal modeling and biophysical simulations to multimodal neuroimaging data in patients with temporal lobe epilepsy and healthy controls. They demonstrate robust increases in brain‐wide excitability in patients, show associations to electroclinical and cognitive measures, as well as to disease progression. Findings are shown in two independent datasets, suggesting replicability.

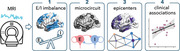

## 1 Introduction

The balance between excitatory and inhibitory (E/I) signaling is a key principle of neuronal dynamics and cortical circuit function,^[^ ^1^, ^2^ ^]^ and plays a crucial role in typical neurodevelopment and the emergence of large‐scale network coordination.^[^ ^3^, ^4^ ^]^ Conversely, imbalances in E/I have been implicated in numerous neurodevelopmental conditions.^[^ ^5^, ^6^, ^7^, ^8^ ^]^ In particular, epilepsy constitutes a prototype condition of E/I imbalance. Here, E/I imbalances across different brain systems result in spontaneous seizures as well as interictal epileptic phenomena, and can also impart cognitive and psychosocial consequences in patients.^[^ ^9^, ^10^, ^11^ ^]^ Although the pathophysiological mechanisms by which structural and functional brain alternations in epilepsy^[^ ^12^, ^13^, ^14^ ^]^ cause epileptogenic events remain incompletely understood, E/I imbalance emerging from localized as well as distributed networks likely acts as a driver.^[^ ^15^, ^16^ ^]^ In temporal lobe epilepsy (TLE), the most common pharmaco‐resistant focal epilepsy in adults, E/I imbalance is thought to originate primarily from temporolimbic circuits.^[^ ^17^, ^18^ ^]^ However, insights into the role of E/I dysfunction in TLE stem mainly from experimental studies in animal models and ex vivo human tissue samples. In vivo investigations in living human brains remain scarce thus far because of the limited availability of robust E/I biomarkers that are non‐invasive, applicable in humans, and measurable at a large scale.

Functional magnetic resonance imaging (fMRI) provides a unique window into localized and macroscale functional properties in the living human brain.^[^ ^19^, ^20^ ^]^ More recently, advances in fMRI acquisition, processing, and signal modeling have permitted to approximate the E/I ratio with high spatial specificity and biophysical plausibility.^[^ ^5^, ^21^ ^]^ A recent study has proposed the Hurst exponent, a statistical descriptor of the spectral properties of neural time series, as an in vivo MRI marker of the synaptic E/I ratio.^[^ ^5^ ^]^ The Hurst exponent quantifies fractal properties of neural signal by measuring temporal autocorrelation processes within the signal.^[^ ^22^, ^23^ ^]^ In silico modeling of BOLD data has robustly demonstrated an association between the E/I ratio and the Hurst exponent,^[^ ^5^ ^]^ showing that in recurrent neural networks in which excitatory and inhibitory neurons interact, specifically enhancing the excitability of only excitatory neuronal populations (i.e., increasing the overall neuronal E/I ratio) leads to measurable decreases in the Hurst exponent. This result implies that changes in the Hurst exponent in neural time series can be interpreted as a shift in synaptic E/I ratio. Additionally, in vivo chemogenetic experiments in mice have corroborated the in silico findings, showing a reduction in the Hurst exponent via enhanced excitability of pyramidal neurons in the prefrontal cortex.^[^ ^5^ ^]^ Altogether, these observations underscore the utility of the Hurst exponent as an in vivo index of synaptic E/I balance, and highlight its potential for large‐scale investigations of E/I function in human TLE patients.

The human brain, and particularly the neocortex, is organized hierarchically^[^ ^24^ ^]^: cortical neurons assemble locally into microscale circuits that interconnect to form nodes, which in turn assemble to constitute macroscale networks. To gain a deeper understanding of the complex interplay between brain activity and pathophysiological processes, models can provide more biologically plausible insights by incorporating heterogeneity of local neural dynamics based on empirical data.^[^ ^25^ ^]^ In particular, neural mass model governed by anatomical and functional properties can robustly simulate interregional intrinsic functional connectivity from structural connectivity in healthy individuals.^[^ ^26^, ^27^ ^]^ Moreover, model inversion techniques allow for the estimation of region‐specific microcircuit parameters, such as the recurrent connection strength and external subcortical inputs. As biophysical models provide circuit mechanisms underlying physiological (dys‐)functions, their synaptic‐level detail enables the inference of large‐scale E/I variations. neural mass model‐based whole‐brain modeling has proven effective in simulating aberrant dynamics of excitatory and inhibitory neuronal subpopulations, and revealing their specific associations with abnormal accumulations of pathological deposits in neurodegenerative conditions.^[^ ^28^ ^]^ This computational approach opens the possibility of identifying the contribution of circuit‐level alternations of excitation and inhibition to local functional imbalances in patients with TLE.

Seizures have been established to increase markers of excitability, such as glutamate.^[^ ^29^, ^30^, ^31^ ^]^ Furthermore, TLE has consistently been associated with disruptions in glutamatergic and GABAergic circuits,^[^ ^32^, ^33^ ^]^ potentially contributing to the genesis or maintenance of seizure activity. Excessive metabolic activation resulting from disrupted balance in these systems may, in turn, promote excitotoxicity, epileptogenicity, and cell death.^[^ ^34^ ^]^ Ultimately, this process may lead to rapid seizure spread and an extension of the epileptogenic networks, affecting both seizure‐generating and contralateral target regions.^[^ ^35^, ^36^, ^37^, ^38^ ^]^ Despite growing evidence of progressive cortical atrophy in intractable TLE,^[^ ^39^, ^40^, ^41^ ^]^ it remains unclear whether this condition is associated with progressive E/I imbalance. Investigating whether alternations in the Hurst exponent are more pronounced in patients with longer duration of illness could provide new insights into the progression of dysfunction in TLE. Clinically, this raises the possibility that the Hurst exponent could serve as a novel marker for TLE diagnosis and disease staging. In this context, longitudinal designs provide an opportunity to infer causality between seizures and E/I imbalance.^[^ ^39^, ^40^ ^]^ Moreover, such designs can control for aging effects and inter‐subject variability, thereby increasing sensitivity to detect subtle alternations. In addition to experiencing seizures, TLE patients are also affected by cognitive, psychological, and social impairment. Up to 80% of patients demonstrate impairments in at least one cognitive domain—most frequently memory, executive, and language function.^[^ ^42^, ^43^, ^44^, ^45^ ^]^ In a subset of patients, these impairments have been shown to be progressive in nature.^[^ ^46^, ^47^, ^48^ ^]^ Despite the high prevalence of cognitive dysfunction in TLE, there is significant variability in the severity of impairments observed across patients. For example, patients with generalized cognitive impairment demonstrate widespread cortical thinning and diffuse white matter compromise,^[^ ^43^, ^48^, ^49^ ^]^ whereas those with intact cognitive profiles have minimal structural alternations. Emerging evidence suggests that cognitive impairment in TLE is also determined by damage to functional connectivity within the medial temporal lobe.^[^ ^14^, ^50^ ^]^ To date, however, no studies have explored the extent to which E/I alternations can predict cognitive impairments in TLE.

In this study, we profiled cortical E/I imbalance patterns in pharmaco‐resistant TLE patients and determined their associations with microcircuit perturbations and clinical presentations. We derived the region‐wise Hurst exponent from resting‐state fMRI time series as an E/I ratio proxy and compared this metric between TLE patients and matched healthy controls. Subsequently, we employed biophysical computational simulations to elucidate microcircuit‐level mechanisms underlying macroscale E/I imbalance across the brain. Additionally, we explored associations between E/I ratio alternations and brain perfusion alterations as well as electroclinical parameters. Finally, to demonstrate clinical relevance, we assessed the progression of E/I imbalance in our patient cohort, and its relation to clinical scores of disease severity and cognitive function using both cross‐sectional and longitudinal designs. The reproducibility of our findings was verified in an independent validation dataset.

## 2 Results

We analyzed two independent datasets with multimodal MRI data (i.e., structural, diffusion, and resting‐state fMRI). The discovery dataset *MICA‐MICs*, collected at Montreal Neurological Institute‐Hospital,^[^ ^51^ ^]^ included 80 participants (40 healthy controls and 40 pharmaco‐resistant TLE). The replication dataset *EpiC* included 60 participants (30 healthy controls and 30 pharmaco‐resistant TLE) from Universidad Nacional Autónoma de Mexico.^[^ ^45^, ^48^ ^]^ TLE diagnosis was determined according to the classification of the ILAE.^[^ ^52^ ^]^ Details of subject inclusion criteria are provided in the Experimental Section. Site‐specific demographical and clinical information are shown in **Table** **1**. All participants were aged between 18 and 63 years, with no significant group differences observed in age or sex.

**Table 1:** Demographic and clinical information.

	Discovery dataset (*MICA‐MICs*)			Replication dataset (*EpiC*)		
	Controls (*n* = 40)	TLE (*n* = 40)	*P*‐value	Controls (*n* = 30)	TLE (*n* = 30)	*P*‐value
Age, years	34.25 ± 3.98 (28–44)	35.80 ± 11.04 (18–63)	0.406^a)^	31.83 ± 11.35 (18–57)	30.87 ± 11.46 (18–58)	0.744^a)^
Sex, male/female	19/21	17/23	0.653^b)^	11/19	10/20	0.787^b)^
Seizure focus, L/R	–	27/13	–	–	18/12	–
Age at seizure Onset, years	–	21.80 ± 11.24 (6–60)	–	–	14.96 ± 10.51 (0.7–40)	–
Duration of Epilepsy, years	–	14.00 ± 11.27 (1–45)	–	–	15.58±13.14 (1–49)	–
ASMs	–	2.28 ± 0.91 (1–5)	–	–	1.53 ± 0.63 (1–3)^d)^	–
Surgery (Engel I)	–	16 (11)^c)^	–	–	–	–
HS, *n* (%)	–	21 (52.50)	–	–	24 (80.00)	–

Age, age at seizure onset, and duration of epilepsy are presented as mean ± SD years.

^a)^ Two‐sample *t*‐test;

^b)^ Chi‐square test;

^c)^ Engel I: seizure‐free, *i.e*., Class I postsurgical outcome in Engel's classification;

^d)^ Information available in 26 TLE patients.

TLE = temporal lobe epilepsy; L = left; R = right; ASMs = antiseizure medications; HS = hippocampal sclerosis.

### 2.1 Hurst Exponent Alternations in TLE

**Figure 1:**
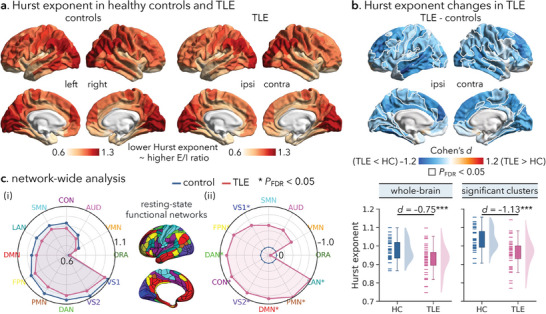
Hurst exponent alterations in TLE patients relative to healthy controls. (a) Mean regional patterns of the Hurst exponent of resting‐state fMRI time series in healthy controls and TLE patients: the lower the Hurst exponent, the higher the excitation/inhibition (E/I) ratio.^[^ ^5^, ^54^ ^]^ (b) Top: Statistical map of TLE‐control difference in regional Hurst exponent, effect size as Cohen's *d*. Significant regions, corrected for multiple comparisons using the false discovery rate procedure (*P*_FDR_ < 0.05), are surrounded by solid white outlines. Higher/lower Hurst exponent values in TLE are shown by warm/cold colors, corresponding to a lower/higher E/I ratio. Bottom: Participant‐specific mean Hurst exponent values across the whole brain and in statistically significant regions, respectively. Boxes represent the interquartile range (IQR), and the lower and upper boundaries of the box correspond to the 25th and 75th percentiles. The whiskers extend to the minimum and maximum values within 1.5 × IQR, and data points beyond the whiskers are displayed as outliers. Each small line represents a participant. (c) (i) Distribution of the average Hurst exponent in 12 large‐scale functional networks in healthy control and TLE groups, respectively. (ii) Distribution of TLE‐control differences in the Hurst exponent with respect to each network (*P*_FDR_ < 0.05).^[^ ^59^ ^] ***^ *p* < 0.001; HC = healthy controls; TLE = temporal lobe epilepsy; ipsi = ipsilateral; contra = contralateral; AUD = auditory network; CON = cingulo‐opercular network; DAN = dorsal attention network; DMN = default mode network; FPN = fronto‐parietal network; LAN = language network; ORA = orbito‐affective network; PMN/VMN = posterior/ventral multimodal network; SMN = somatomotor network; VS1/VS2 = primary/secondary visual network.

We calculated the region‐wise Hurst exponent value, a metric that is mathematically related to the 1/f exponent of neural signals^[^ ^21^, ^53^ ^]^ and commonly used as an index of the E/I ratio. As previously described,^[^ ^5^, ^54^ ^]^ resting‐state fMRI time series were modeled as multivariate fractionally integrated processes, and the Hurst exponent was estimated via the univariate maximum likelihood method and discrete wavelet transform.^[^ ^5^ ^]^ As such, an elevated E/I ratio would manifest in a lower Hurst exponent value. In healthy control and TLE groups reported here, interregional variations in the Hurst exponent values exhibited hierarchical gradients, with the highest values observed in the sensory regions, intermediate values in the association regions, and lowest values in the paralimbic regions (**Figure** **1**a,c). This pattern of sensory‐fugal distinction was confirmed by a significant spatial correlation with the principal axis of cytoarchitectural differentiation (*rho* = −0.41, *P*_spin_ = 0.044; Figure S1a, Supporting Information), previously established through analysis of myelin‐sensitive MRI.^[^ ^55^ ^]^ To further contextualize our regional pattern of the Hurst exponent, we correlated it with morphometric and molecular markers of phylogenetic cortical differentiation.^[^ ^56^ ^]^ We found that at the surface level, the Hurst exponent positively correlated with intracortical myelination (*rho* = 0.37, *P*_spin_ = 0.066) and gene expression gradient (*rho* = 0.60, *P*_spin_ = 0.002),^[^ ^57^ ^]^ while negatively correlated with cortical thickness (*rho* = −0.51, *P*_spin_ = 0.007) and neurotransmitter receptor gradient (*rho* = −0.46, *P*_spin_ = 0.009^[^ ^58^ ^]^; Figure S1b, Supporting Information).

Importantly, in comparison to healthy controls, TLE patients exhibited marked decreases in the Hurst exponent both at global and local levels. Specifically, TLE patients had a significantly lower grand average Hurst exponent value across the entire brain relative to healthy controls (Cohen's *d* = −0.75, *p* < 0.001; male/female, *d* = −0.80/−0.72, *p* = 0.012/0.011; Figure 1b). Surface‐based analysis revealed significant decreases in the Hurst exponent values in 156 out of 360 cortical regions in TLE compared to healthy controls following correction for multiple comparisons at a false discovery rate of *P*_FDR_ < 0.05 (Figure 1b; male vs female TLE, *rho* = 0.28, *P*_spin_ = 0.002, Figure S2, Supporting Information). These mostly affected the lateral inferior, middle, and superior temporal lobes, dorsolateral and dorsomedial prefrontal cortex, fusiform gyrus, precuneus, and the occipital cortex bilaterally, together with the ipsilateral postcentral gyrus, with effect sizes ranging from medium to large (*d* = −0.46 to −1.22, mean *d* ± SD = −0.64 ± 0.07). As for the subcortical regions, the bilateral caudate and thalamus were most affected (*d* = −0.58 to −0.67, mean *d* ± SD = −0.63 ± 0.02; Figure S3, Supporting Information), in addition to the ipsilateral hippocampal (*d* = −0.62). These findings were verified in a subgroup with histologically confirmed mesiotemporal sclerosis and post‐surgical seizure freedom at a 1‐year follow‐up (i.e., Engel I, *n* = 11; whole‐brain/significant clusters/subcortex: *d* = −1.56/−1.94/−1.18, *p* < 0.001; Figure S4, Supporting Information). When comparing TLE patients with (*n* = 21, 52.50%) and without (*n* = 19, 47.50%) hippocampal sclerosis, we observed a trend toward lower Hurst exponent values in the former subgroup (whole‐brain/significant clusters/subcortex: *d* = −0.52/−0.56/−0.66, *p* = 0.054/0.042/0.021). Finally, to assess whether distributed patterns of the Hurst exponent differences were more pronounced in specific brain systems, we used two brain system definitions: 1) intrinsic functional networks defined by Ji et al.^[^ ^59^ ^]^ and Yeo et al.^[^ ^60^ ^]^; and 2) a cytoarchitectonic classification of human cortex based on von Economo atlas.^[^ ^61^ ^]^ In terms of intrinsic networks, significant TLE‐control differences were seen in the transmodal association system, including the default mode, frontoparietal, and dorsal/ventral attention networks, as well as the unimodal visual system (*P*_FDR_ < 0.05; Figure 1c; Figure S5, Supporting Information). In terms of cytoarchitectonic classes, the association, limbic, and sensory cytoarchitectonic classes were the most affected (*P*_FDR_ < 0.05; Figure S5, Supporting Information). To ensure that our results were not related to spurious features, we assessed the degree of head motion of each individual during resting‐state fMRI scans based on framewise displacement.^[^ ^62^ ^]^ Notably, between‐group differences in the global average (*d* = −0.47, *p* = 0.020) and local value of the Hurst exponent (mean ± SD *d* = −0.58 ± 0.07; Figure S6, Supporting Information) were robust when additionally controlling for individual‐wise mean framewise displacement, suggesting no marked influence of head motion.

### 2.2 Microcircuit Parameter Alterations in TLE

**Figure 2:**
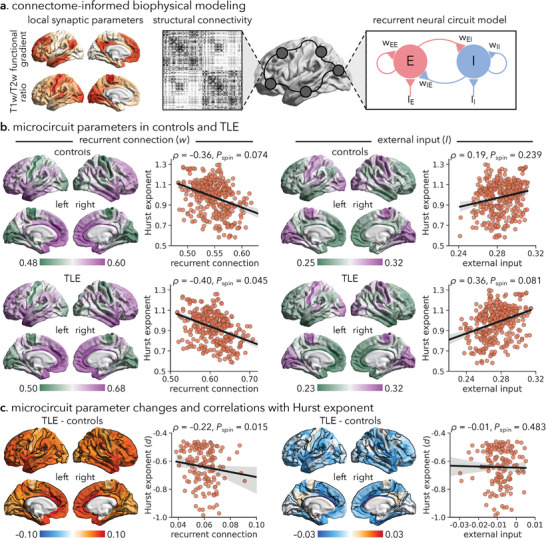
Microcircuit parameter alterations in TLE and associations with Hurst exponent alternations. (a) Schematic of the parametric mean‐filed model (pMFM) to estimate region‐specific microcircuit parameters (*i.e*., recurrent connection strength *w*, external input *I*) from the structural connectome (based on diffusion MRI tractography).^[^ ^26^ ^]^ The pMFM is a neural mass model derived from the mean‐field reduction of spiking neuronal network models.^[^ ^65^ ^]^ The pMFM consists of differential equations at each brain region that govern the neural dynamics of excitatory (“E”) and inhibitory (“I”) neuronal populations. Red/blue circles represent excitatory/inhibitory connections. For example, *w*_EI_ indicates the strength of connection from the excitatory population to the inhibitory population. The regional microcircuit parameters (*w* and *I*) of the pMFM vary across brain regions and are parameterized by a linear combination of resting‐state functional connectivity gradient and T1w/T2w ratio estimate maps. (b) Regional microcircuit parameters of healthy controls (*top*) and TLE patients (*bottom*) and associations with regional Hurst exponent values at the surface level. (c) Regional differences in microcircuit parameters (TLE‐control) and relations to the effect sizes (i.e., Cohen's *d*) of Hurst exponent alterations, constrained to brain regions showing significant between‐group differences in Figure 1b (solid black outlines). The statistical significance (*i.e*., *P*_spin_) of spatial correlation between cortical maps is determined via spin permutation tests (5000 iterations).^[^ ^66^ ^]^

Next, we cross‐validated our findings and explored circuit mechanisms underlying disruptions of E/I balance in TLE using a parametric mean‐field model (pMFM).^[^ ^26^ ^]^ In the biophysically‐based model, local neuronal dynamics were simulated through a set of simplified nonlinear stochastic differential equations (see Experimental Section) by linking ensembles of local neuronal masses with diffusion‐derived structural connectivity.^[^ ^63^ ^]^ The pMFM iteratively tuned its parameters (i.e., recurrent connection strength *w*, external input current *I*, noise *σ*, and a constant *G*) to simulate neural signals that were maximally similar to the empirical data. Adopting a recent framework,^[^ ^26^ ^]^ *w*, *I*, and *σ* varied across brain regions and were parameterized by a linear combination of local structural (i.e., intracortical myelination) and functional (i.e., resting‐state functional connectivity gradient) properties (**Figure** **2**a). More specifically, for each group (healthy control or TLE), the 40 participants were randomly subdivided into the training (*n* = 15), validation (*n* = 15), and test (*n* = 10) sets. Group‐averaged structural connectivity, static functional connectivity (FC), and time‐varying functional connectivity dynamics (FCD) were computed separately for each set. Next, the 250 candidate parameter sets (*w*, *I*, *σ*, and *G*) were generated from the training set of each group using the CMA‐ES algorithm and evaluated in the validation set.^[^ ^64^ ^]^ The top ten parameter sets from the validation set were then evaluated in the test set. To ensure stability, the split of participants into training, validation, and test sets was repeated five times. Finally, the pMFM parameters based on the best fit (with the lowest cost between simulated and empirical FC and FCD matrices; see Experimental Section) from the test set were averaged across these five splits, yielding the representative set of parameters for each group.

In agreement with prior work,^[^ ^26^ ^]^ in both cohorts, recurrent connection strength (*w*) gradually increased from primary sensory/motor cortices to high‐order association cortices in the brain, reaching its highest value in the default mode network. On the other hand, external input (*I*) smoothly decreased along the unimodal‐transmodal hierarchy (Figure 2b; Figure S7, Supporting Information). By correlating regional variations in microcircuit parameters with the Hurst exponent, we find that recurrent connection strength (*w*) appears to be stronger in brain regions with a lower Hurst exponent (i.e., higher E/I ratio) (healthy controls: *rho* = −0.36, *P*_spin_ = 0.074; TLE: *rho* = −0.40, *P*_spin_ = 0.045; Figure 2b). On the other hand, regions with higher local external input (*I*) tend to have a relatively higher Hurst exponent (i.e., lower E/I ratio) (healthy controls: *rho* = 0.19, *P*_spin_ = 0.239; TLE: *rho* = 0.36, *P*_spin_ = 0.081; Figure 2b). Moreover, compared to healthy controls, TLE patients showed increases in recurrent connection strength (*w*) as well as decreases in external input (*I*). TLE‐related decreases in the Hurst exponent were enriched in regions with the greatest effects of increasing recurrent connection strength (*rho* = −0.22, *P*_spin_ = 0.015). By contrast, no significant correlation was found between the degree of the Hurst exponent changes and regional alternations in external input current (*rho* = −0.01, *P*_spin_ = 0.483; Figure 2c).

### 2.3 Network‐Level Effects of Hurst Exponent Alternations

**Figure 3:**
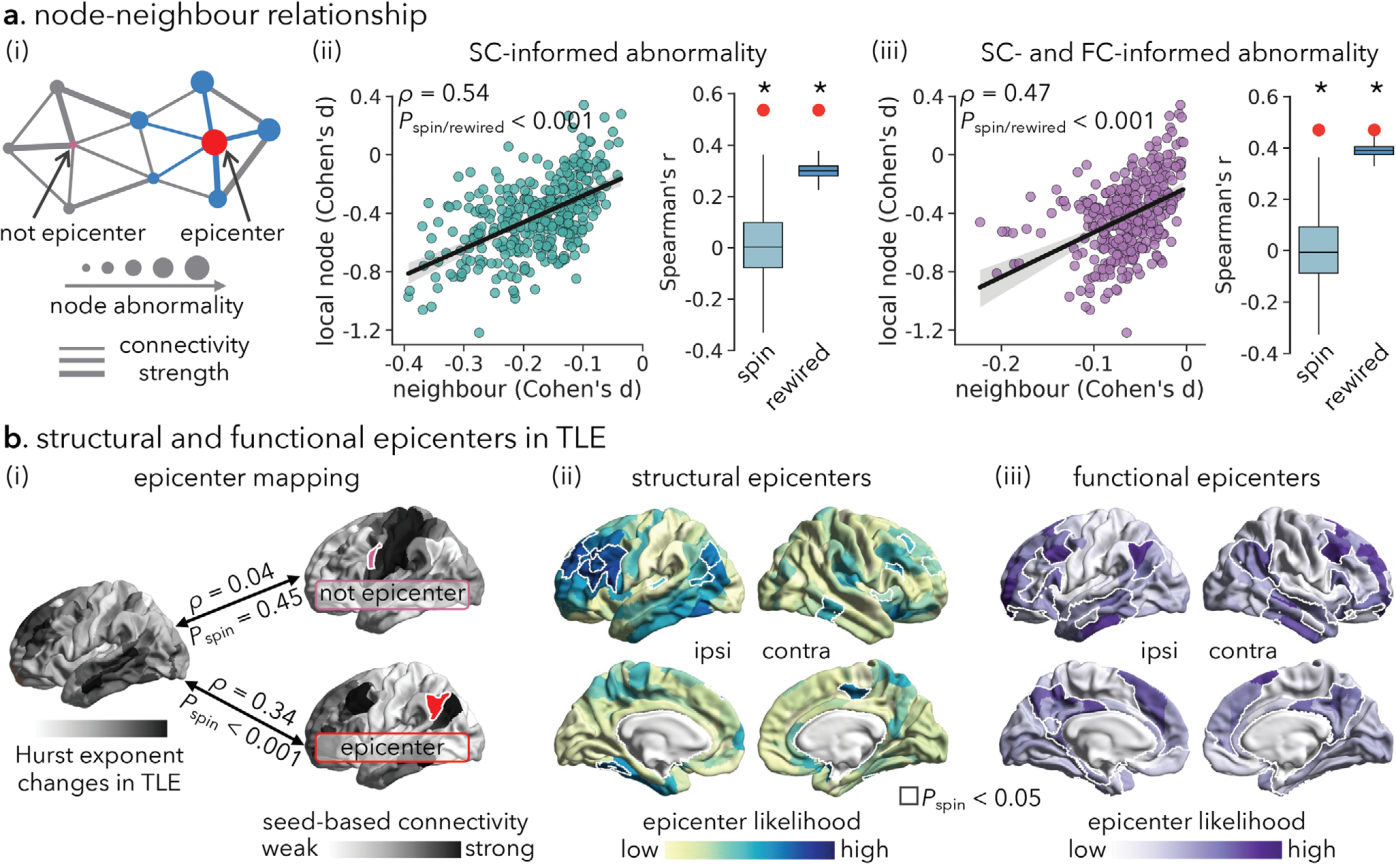
Network‐based spreading of Hurst exponent alternations. (a) (i) Schematic of structural (SC) or functional (FC) connectivity informing TLE‐related Hurst exponent alternations. (ii, iii) Correlations between node alternation and SC‐/FC‐informed mean neighbor alternation. Box plots: The observed Spearman correlation coefficients (shown as red circles) between node and neighbor alternation patterns are compared against two null models: (1) “spin tests” by generating 5000 surrogate maps through randomly rotating region‐level Cohen's *d* values (light blue boxes); (2) “rewired tests” by randomly rewiring edges while preserving the nodal degree and edge length distributions of the empirical structural connectome 1000 times (dark blue boxes). Boxes represent the interquartile range, with the median shown as an inside line, and the lower and upper boundaries of the box correspond to the 25th and 75th percentiles. ^*^ *p* < 0.001. (b) Epicenters of TLE‐related Hurst exponent alternations. (i) A node whose SC or FC pattern across the entire cortex strongly correlated with TLE‐related Hurst exponent alternation map (see Figure 1b) is considered a likely “epicenter”. Epicenter likelihood is defined as the correlation coefficient between the two maps. (ii, iii) SC‐ and FC‐informed epicenter likelihood maps of TLE‐related Hurst exponent alternations, where the most likely epicenters, assessed using spin permutation tests (5000 iterations, and *P*_spin_ < 0.05), are surrounded by solid white outlines. ipsi = ipsilateral; contra = contralateral.

Local vulnerability interacts with brain network architecture to shape disease pathology and spread.^[^ ^67^, ^68^, ^69^, ^70^ ^]^ Here, we assessed the extent to which TLE‐related alternations in the Hurst exponent exhibited network effects. For each node, we computed the mean Hurst exponent changes (i.e., mean Cohen's *d*) of its connected neighbors, weighted by streamline density estimated using diffusion MRI and functional connectivity strength estimated using resting‐state fMRI (**Figure** **3**a). To ensure that connectivity estimates reflect the typical connectomes prior to disease onset and deafferentation, we estimated group‐level structural and functional connectivity matrices in a sample of 100 unrelated healthy young adults from the Human Connectome Project (HCP).^[^ ^71^ ^]^ We observed a strong association between the alternation in the Hurst exponent of a node and the mean alternation of its structurally connected neighbors (*rho* = 0.54, *P*_spin_/*P*_rewired_ < 0.001; Figure 3a). Similarly, there was a comparable association between the alternation in the Hurst exponent of a node and the mean alternation of its functionally connected neighbors (*rho* = 0.47, *P*_spin_/*P*_rewired_ < 0.001; Figure 3a). That is, pathology in a brain region is closely correlated with greater exposure to pathology in anatomically and/or functionally connected regions.

Having observed that network architecture reflects TLE‐related Hurst exponent alternation, we then examined which brain regions likely act as putative disease epicenters. As previously introduced,^[^ ^41^, ^70^ ^]^ we defined an epicenter as a node that had a structural and functional connectivity profile that spatially resembled the whole‐brain pattern of TLE‐related Hurst exponent alterations (Figure 3b). This measure identifies “disorder hubs”—regions that are both vulnerable to disorder‐specific alternations but also embedded in a highly atypical network cluster. Nodes were ranked in descending order based on their correlation coefficients. Empirical epicenter likelihood rankings were compared with rankings estimated from spatial autocorrelation‐preserving null models (5000 iterations). The highest ranked epicenters were in prefrontal and temporal cortices, including dorsolateral prefrontal, and middle and inferior temporal cortices (*P*_spin_ < 0.05; Figure 3b). As for subcortical areas, bilateral nucleus accumbens (*P*_spin_ < 0.05), bilateral caudate and thalamus (*P*_spin_ < 0.05), and ipsilateral hippocampus (*P*_spin_ = 0.063) emerged as potential structural and functional disease epicenters, respectively (Figure S8, Supporting Information).

### 2.4 Clinical Utility of the Hurst Exponent

#### 2.4.1 Associations with Clinical and Cognitive Variables

**Figure 4:**
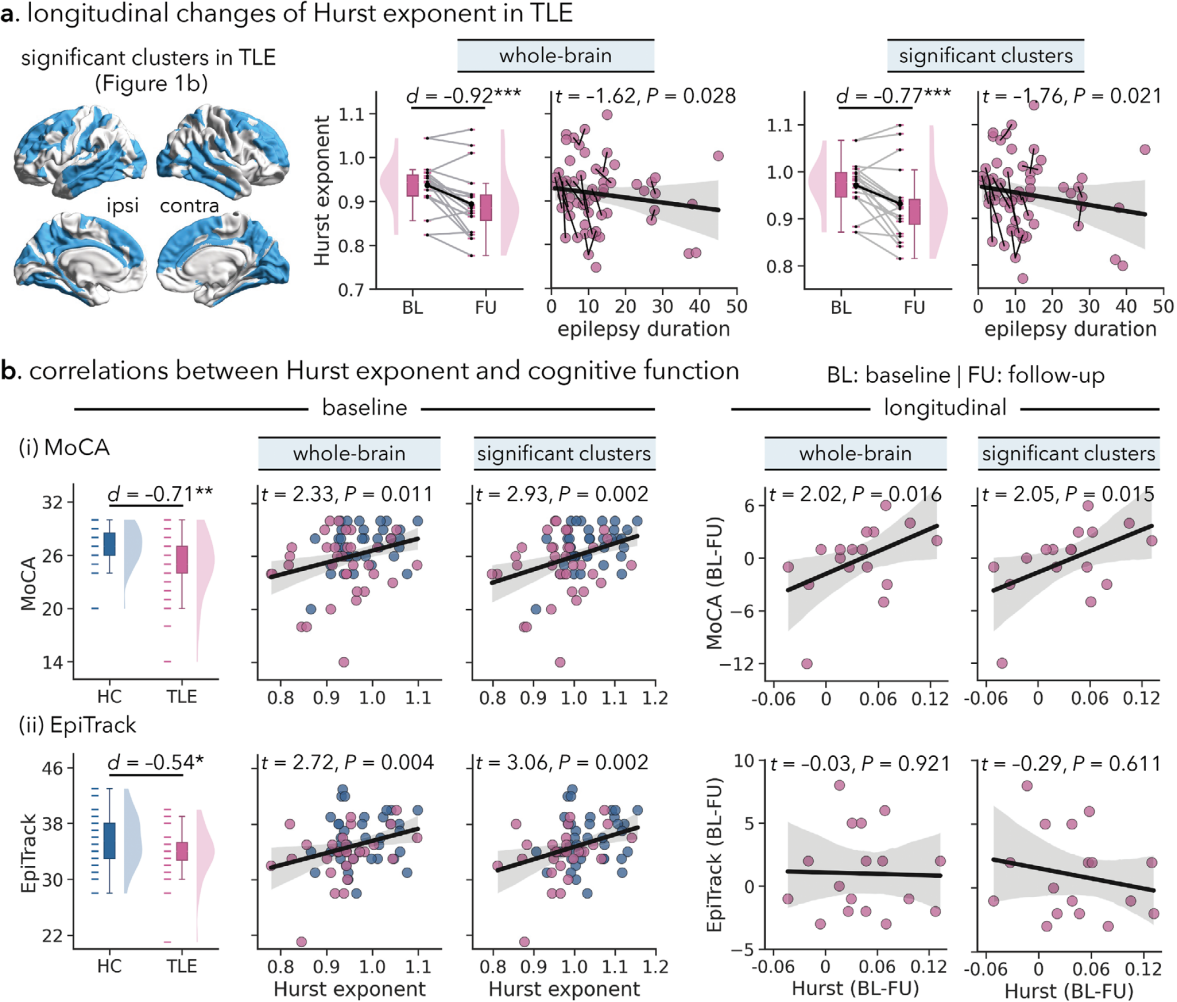
Associations of the Hurst exponent with clinical characteristics and behavioral assessments. (a) Error bar plots: longitudinal alternations in the Hurst exponent in TLE patients. Spaghetti plots: associations between epilepsy duration and the Hurst exponent across both baseline and follow‐up time scans. (b) Left: MoCA and EpiTrack scores in TLE patients and healthy controls at baseline. Middle: Associations of the Hurst exponent and MoCA, EpiTrack scores at baseline. Right: Associations of longitudinal alternations in the Hurst exponent and MoCA, EpiTrack scores in TLE patients. ^*^ *p* < 0.05; ^**^ *p* < 0.01, ^***^ *p* < 0.001. HC = healthy controls; TLE = temporal lobe epilepsy; MoCA = Montreal Cognitive Assessment; BL = baseline; FU = follow‐up.

Associations between Hurst exponent alternations and clinical characteristics of disease severity were assessed in TLE patients. A longer disease duration was negatively correlated with the Hurst exponent (whole‐brain: *t* = −1.62, *p* = 0.028; significant clusters: *t* = −1.76, *P* = 0.021; **Figure** **4**a), indicating relatively lower Hurst exponent in patients with long‐standing TLE. There were also negative correlations between the Hurst exponent and the number of electroclinical seizures captured during hospitalization (*r* = −0.38, *p* = 0.009; *r* = −0.36, *p* = 0.013; Figure S9, Supporting Information), such that more frequent epileptic seizures were associated with lower Hurst exponent. Similarly, we found significantly lower Hurst exponent values in TLE patients with prevalent interictal epileptiform discharges (IEDs) compared to those with rare IEDs (*d* = −0.70, *p* = 0.029; *d* = −0.67, *p* = 0.034; Figure S10, Supporting Information). No significant correlations were found between the Hurst exponent and age at seizure onset or the number of antiepileptic drugs (*p* > 0.150). To further understand the neurophysiological substrate of TLE‐related alternations in the E/I ratio, we analyzed cerebral blood flow measured by pseudo‐continuous arterial spin labeling (ASL) MRI in patients with TLE. We found significantly lower cortical perfusion (or hypoperfusion) in brain regions (*rho* = 0.41, *P*_spin_ < 0.001) or patients with lower Hurst exponent values (whole‐brain: *r* = 0.40, *p* < 0.001; significant clusters: *r* = 0.43, *p* < 0.001; Figure S11, Supporting Information).

We then explored the associations between the Hurst exponent and cognitive function in TLE patients both cross‐sectionally and longitudinally. As previously reported,^[^ ^72^ ^]^ at baseline, TLE patients showed markedly poorer performance than healthy controls in general cognitive functioning as measured by the MoCA test^[^ ^73^ ^]^ (*d* = −0.71, *p* = 0.002), as well as in attention and executive functions as measured by the EpiTrack test^[^ ^74^ ^]^ (*d* = −0.54, *p* = 0.017; Figure 4b). The Hurst exponent positively correlated with MoCA scores (whole‐brain: *r* = 0.27, *t* = 2.33, *p* = 0.011; significant clusters: *r* = 0.33, *t* = 2.93, *p* = 0.002), as well as EpiTrack scores (*r* = 0.33, *t* = 2.72, *p* = 0.004; *r* = 0.36, *t* = 3.06, *p* = 0.002; Figure 4b), indicating more marked cognitive impairment in patients with lower Hurst exponent values. Moreover, by analyzing for whom longitudinal data was available, we found that the Hurst exponent progressively decreased over a mean interscan interval of 1.8 years (whole‐brain: *d* = −0.92, *p* < 0.001; significant clusters: *d* = −0.77, *p* < 0.001; Figure 4a), and correlated with the progressive decline in MoCA scores (*t* = 2.02, *p* = 0.016; *t* = 2.05, *p* = 0.015; Figure 4b). There were no significant associations between longitudinal alterations in the Hurst exponent and the number of antiseizure drugs (whole‐brain: *t* = −0.82, *p* = 0.424; significant clusters: *t* = −1.05, *p* = 0.307), indicating that the overall number of antiseizure drugs does not substantially affect the progressive decreases in E/I ratio. Patients were further divided into two subgroups based on the cognitive impact of their antiseizure medications: those on medications with more cognitive side effects and those on medications with fewer or no side effects. No significant differences were found in the longitudinal changes in Hurst exponent values between the two groups (whole‐brain: *d* = 0.15, *p* = 0.760; significant clusters: *d* = −0.01, *p* = 0.975; see Table S2, Supporting Information for further details).

#### 2.4.2 Case‐Control Classification

Using a supervised machine learning algorithm (SVM)^[^ ^75^ ^]^ with 4‐fold cross‐validation, Hurst exponent‐informed classifier achieved the highest performance in discriminating patients from healthy controls using 14 subcortical regions and the top 10% (i.e., 36) of cortical areas showing TLE‐control differences as features (accuracy: mean ± SD = 72.4% ± 3.4% [57.5%–82.5%]; AUC: mean ± SD = 0.78 ± 0.03 [0.68–0.86]). Permutation tests with 1000 randomly shuffled participant labels indicated that the classifier performance exceeded chance levels (accuracy: *p* = 0.008; AUC: *p* = 0.001). Classifiers based on the top 20, 30, 40, and 50% of cortical areas showing TLE‐control differences demonstrated comparable performance (all *p* < 0.001; mean accuracy = 72.0%/71.6%/71.3%/71.0%; mean AUC = 0.77/0.77/0.76/0.76).

#### 2.4.3 Replication Analysis

**Figure 5:**
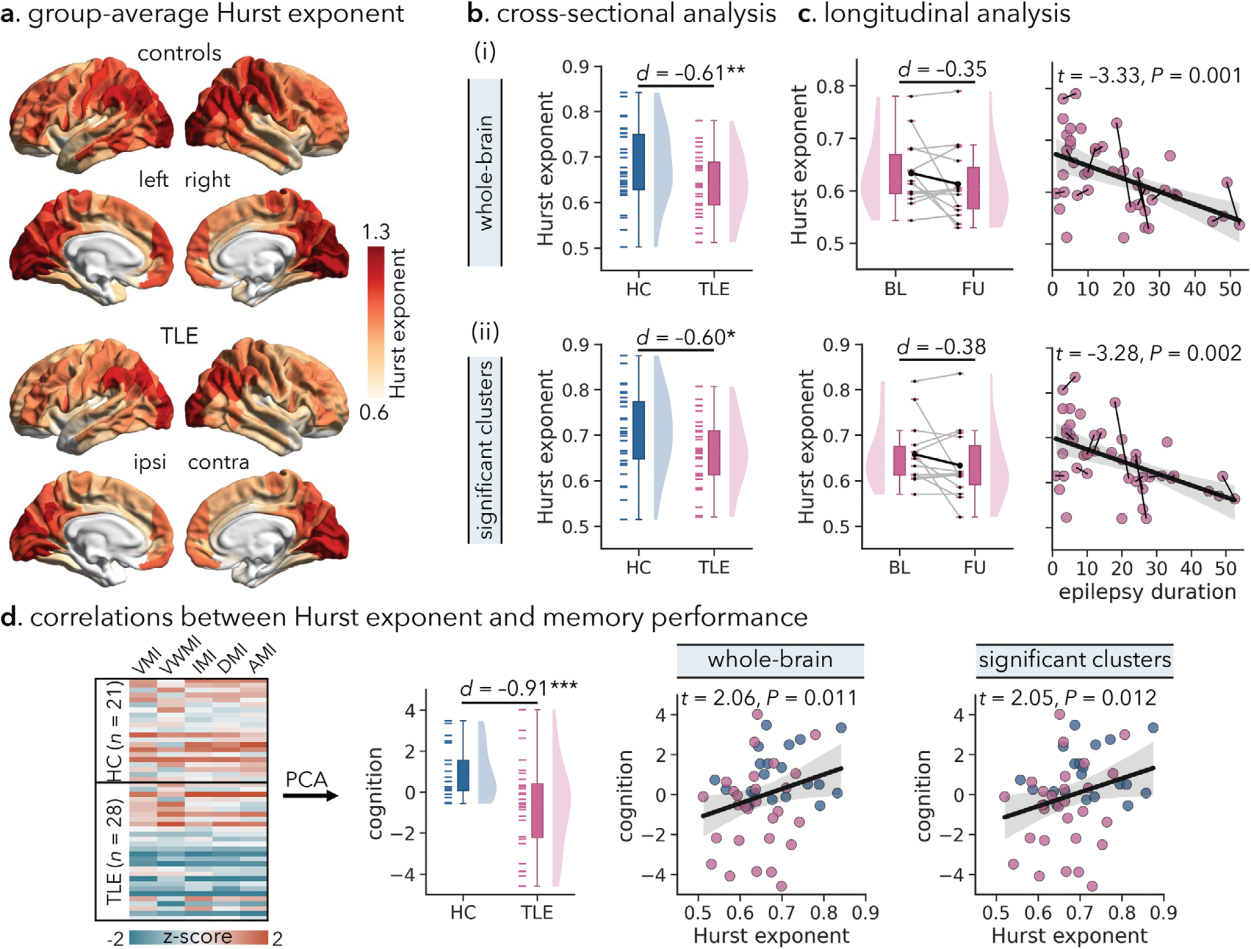
Replication analysis of the Hurst exponent alterations in the *EpiC* dataset. (a) Group‐averaged Hurst exponent in healthy control and TLE groups. (b) TLE‐control differences in the average Hurst exponent across the entire brain (i) and significant clusters (ii) identified from the discovery dataset (see Figure 1b). (c) Error bar plots: Longitudinal alternations in the Hurst exponent in TLE patients. Spaghetti plots: Associations between epilepsy duration and the Hurst exponent across baseline and follow‐up time points. (d) Relationships between individual memory performance and the Hurst exponent at baseline. Error bar plots: Between‐group differences in the overall memory performance (i.e., PC1 loading score) determined through a principal component analysis on various memory tests. Scatter plots: Hurst exponent positively correlated with the PC1 loading score after controlling for age and sex. ^*^ *p* < 0.050; ^**^ *p* < 0.010; ^***^ *p* < 0.001. HC = healthy controls; TLE = temporal lobe epilepsy; BL = baseline; FU = follow‐up; AMI = auditory memory; DMI = delayed memory; IMI = immediate memory; VMI = visual memory; VWMI = visual working memory.

Hurst exponent alternations in TLE were replicable in an independent replication dataset (i.e., EpiC) consisting of 30 pharmaco‐resistant TLE patients and 30 healthy controls. Specifically, in the *EpiC* dataset, the spatial pattern of the Hurst exponent in each group closely resembled that observed in the *MICA‐MICs* dataset, gradually decreasing along the sensory‐fugal axis (*rho* > 0.89, *P*_spin_ < 0.001; **Figure** **5**a). Comparing cohorts in the *EpiC* dataset, TLE patients also exhibited significantly decreased Hurst exponent globally (*d* = −0.61, *p* = 0.011), locally in significant regions identified from the discovery dataset (*d* = −0.60, *p* = 0.012; Figure 5b), and in the subcortex (*d* = −0.47, *p* = 0.038). Region‐wise group differences in the cortical Hurst exponent were also spatially correlated between the two datasets at the surface level (*rho* = 0.20, *P*_spin_ = 0.010). Furthermore, we observed a noticeable trend toward progressive decreases in the Hurst exponent in TLE patients over a mean interscan period of 2.5 years (whole‐brain: *d* = −0.35, *p* = 0.105; significant clusters: *d* = −0.38, *p* = 0.087). A longer epilepsy duration was associated with a greater extent of decreases in the Hurst exponent (*t* = −3.33, *p* = 0.001; *t* = −3.28, *p* = 0.002; Figure 5c). Finally, we replicated the finding of the cognitive relevance of an altered Hurst exponent, with evidence of marked memory deficits in TLE compared to healthy controls (PC1 score: *d* = −0.91, *p* < 0.001), as well as lower Hurst exponent values in TLE with poorer memory performance (*t* = 2.06, *p* = 0.011; *t* = 2.05, *p* = 0.012; Figure 5d).

## 3 Discussion

In this work, we mapped cortical E/I imbalance in patients with pharmaco‐resistant TLE and assessed its relationships with microcircuit‐level dysfunction, electroclinical parameters, and cognitive impairments. We found significant decreases in the Hurst exponent in TLE compared to controls in cortical networks that extended beyond the temporolimbic cortex to frontoparietal and occipital regions, suggesting a shift in E/I balance toward large‐scale network excitation. Leveraging whole‐brain biophysical simulations, we demonstrated that enhancing cortical excitation in TLE reflected atypical increases in recurrent connection strength within the structurally governed connectome. Moreover, mixed cross‐sectional and longitudinal analysis unveiled more marked Hurst exponent decreases in TLE with longer disease duration, more frequent electroclinical seizures, and interictal epileptic spikes, as well as poorer cognitive function. The Hurst exponent showed a progressive decrease at longitudinal follow‐up and correlated with the simultaneous longitudinal worsening of cognitive function in patients. Finally, a supervised model informed by Hurst exponent data discriminated TLE patients from healthy controls with relatively high accuracy. Our findings were replicated in an independent dataset, suggesting generalizability. Taken together, our work provides in vivo support for a cortical E/I imbalance shifting toward excitation in pharmaco‐resistant TLE. These findings enhance our understanding of the interplay between macroscale cortical dysfunction, microcircuit perturbation, and cognitive alterations, potentially informing new treatment strategies targeting E/I mechanisms.

Our study examined the Hurst exponent, an in vivo MRI marker of E/I balance, in TLE. Prior research has indicated that the Hurst exponent in neural time series data closely reflects underlying alterations in the synaptic E/I ratio.^[^ ^5^ ^]^ In recurrent networks, where excitatory and inhibitory neuronal populations interact, the Hurst exponent decreases with increasing network excitability. Although net E/I effects are typically balanced in local circuits in healthy individuals, there are slight variations in the degree of balance across regions. Specifically, in our healthy control group, the Hurst exponent values gradually decreased along the sensory‐fugal hierarchy,^[^ ^55^ ^]^ with the highest values observed in the primary sensory regions with heavy myelination and laminar organization, and the lowest values in paralimbic regions. Increased levels of myelination have been reported to suppress the formation of new axonal tracts and synapses, thus potentially reducing spikes and yielding neural activity with less scale‐free or critical properties.^[^ ^1^, ^76^, ^77^ ^]^ By contrast, lower myelination in frontal and limbic cortices allows for greater functional signal variability and neuronal remodeling at various timescales, facilitating the emergence of diverse functional dynamics.^[^ ^55^, ^78^ ^]^ Importantly, by comparing patients to controls, we observed marked decreases in the Hurst exponent values in the former group across multiple lobes, affecting lateral and medial temporal cortices, frontocentral cortices, hippocampus, and the thalamus. Despite findings being diffuse, they nevertheless point to temporo‐limbic epicenters that are thought to be hallmarks of TLE and to participate in the putative pathophysiological circuit of TLE.^[^ ^70^, ^79^ ^]^ Our findings align with a model^[^ ^80^, ^81^ ^]^ proposing that TLE‐related pathology primarily targets mesiotemporal cortices as well as interconnected regions and hub nodes at the crossroads of multiple brain networks.^[^ ^82^ ^]^ These findings also reinforce our understanding of TLE classically defined focal syndrome—as a system‐level disorder,^[^ ^83^, ^84^, ^85^ ^]^ likely driven by a network of multiple interconnected regions, rather than being solely dependent on the pathological core in the mesiotemporal lobe. While network‐based indices have been instrumental in mapping brain topology,^[^ ^86^ ^]^ particularly large‐scale connectivity gradients, the Hurst exponent offers a complementary and unique perspective by capturing the temporal complexity of neural activity. Unlike gradient‐based metrics, which primarily reflect spatial patterns of functional organization, the Hurst exponent provides insights into long‐range temporal correlations, revealing the stability and adaptability of neural function over time. This ability to characterize the temporal dynamics of excitability and inhibition across brain regions is especially valuable in TLE, where disruptions in E/I balance play a key role in both seizure generation and progression. Our results are also in line with existing in vitro imaging data in TLE demonstrating overall hyper‐excitability of local networks, which may be fostered by atypical excitatory and inhibitory processes at the cellular level.^[^ ^10^ ^]^ Although the current study examined a relatively homogenous cohort of patients with electroclinical features of unilateral TLE, alterations in the Hurst exponent encompassed a bilateral territory. Previous electrophysiology studies have shown that seizures originating in the ipsilateral mesiotemporal region often propagate to the contralateral temporal lobe directly along commissural pathways or indirectly via other regions, such as the frontal lobes.^[^ ^87^ ^]^ Ultimately, cellular and synaptic alterations may occur in both seizure‐generating ipsilateral regions and contralateral zones of propagation.^[^ ^88^ ^]^ In TLE, increased slow waves are characteristic features during the interictal period; higher incidences of slow waves are related to greater volume loss in mesiotemporal structures.^[^ ^89^ ^]^ This suggests a potential link between neuronal death, synaptic loss, and cortical hyperactivity, which warrants future validation using pathology data from patients undergoing surgery. In addition, earlier metabolic studies have shown profound reductions in mGluR5 availability in the temporolimbic cortex in TLE,^[^ ^90^, ^91^ ^]^ likely reflecting receptor internalization or conformational changes driven by excessive extracellular glutamate levels.^[^ ^92^ ^]^ Elevated glutamate levels or impaired neurotransmitter cycling (glutamate–glutamine) in the epileptogenic focus^[^ ^29^, ^93^ ^]^ could, in turn, lead to long‐term potentiation and increase seizure likelihood.^[^ ^94^ ^]^ Future studies that image glutamate in vivo, however, will be needed to establish a causal link with hyperexcitability.

To elucidate how cortical E/I disruptions target hub regions in TLE, we tested the hypothesis that the underlying connectivity profiles of specific brain regions constrain TLE‐related patterns of the Hurst exponent alternation. Data‐driven epicenter mapping revealed that regional alternations in the Hurst exponent implicated both structurally and functionally connected neighbors, suggesting that network architecture serves as a scaffold for the spread of E/I imbalance in TLE. Interestingly, bilateral temporolimbic and frontoparietal regions (precuneus, superior parietal cortex) emerged as putative epicenters. These regions are generally considered densely inter‐connected hubs that are thought to support the integration and broadcasting of signals across different subnetworks.^[^ ^95^ ^]^ Hubs are particularly susceptible to pathology, with mounting evidence showing alternations in cortical morphology and connectivity patterns in TLE.^[^ ^20^, ^70^, ^83^ ^]^ Indeed, fMRI studies in TLE have previously identified decreases in long‐range connections of distributed cortical networks, alongside increases in connectivity in temporolimbic circuits proximal to the seizure focus.^[^ ^96^ ^]^ We complement these findings by highlighting that the temporolimbic and frontoparietal cortices are particularly vulnerable to E/I disturbance and that they, by virtue of their network embedding, may increase disease exposure to connected regions. On the other hand, our epicenter mapping approach revealed differing sensitivities of structural and functional connectivity in detecting TLE‐related pathophysiology. Structural epicenters in TLE converged in superior parietal and prefrontal cortices, whereas functional epicenters preferentially occurred in temporo‐limbic cortices and several subcortical regions. This difference may be attributed to the fact that structural and functional connectivity capture fundamentally different features of brain network organization and are only moderately correlated with each other.^[^ ^97^ ^]^ Specifically, diffusion MRI is sensitive to long‐range fiber bundles and direct monosynaptic structural connectivity. In contrast, resting‐state functional MRI allows for the detection of functional connections even in the absence of direct structural connections, thus lending increased sensitivity to the functional identification of polysynaptic cortical systems.^[^ ^98^, ^99^ ^]^

Deviations from macroscale E/I balance may be related to dysfunctions of neural circuits. Our work quantified the extent of perturbations in cortical microcircuit function in TLE, by leveraging a whole‐brain computational model (pMFM) that is biophysically grounded yet parsimonious.^[^ ^26^ ^]^ In recent work, pMFM has demonstrated the ability to predict functional connectivity from structural connectome with robust accuracy and low parametric complexity.^[^ ^26^ ^]^ In our work, interregional variations in recurrent connection strength and external input current followed sensory‐association hierarchical gradients, and exhibited significant correlations with Hurst exponent maps at the surface level. This suggests a potential link between microcircuit dynamics and cortex‐wide heterogeneity in E/I balance. Importantly, comparing model parameters between patients and healthy controls suggested a diffuse pattern of local microcircuit disruptions, particularly marked in default mode and frontoparietal systems. Directly correlating TLE‐related decreases in the Hurst exponent with microcircuit parameters revealed a unique association with increased recurrent connection strength. Given that excitatory neurons within each selective population are interconnected via strong recurrent glutamatergic synapses,^[^ ^65^, ^100^ ^]^ the impaired recurrent connections we observed may reflect aberrant excitatory synaptic currents within local neuronal subpopulations. Consequently, excessive intrinsic neuronal excitability, increasing coupling between excitatory neurons, results in an elevated E/I ratio.^[^ ^65^, ^100^, ^101^ ^]^ Previous in vivo and in vitro studies have identified aberrant glutamate transmission (glutamate–glutamine recycling),^[^ ^93^, ^102^, ^103^ ^]^ as well as inhibitory interneuron hypofunction (e.g., inefficient GABA synthesis, release, and transport)^[^ ^104^, ^105^, ^106^, ^107^ ^]^ as potential causes of neuronal hyperactivity, favoring recurrent seizure activity and prolonging the epileptiform discharges in epilepsy.^[^ ^32^, ^33^, ^88^ ^]^ Future work is warranted to more precisely delineate the contributions from excitatory and inhibitory subpopulation functions toward network hyperexcitability, particularly given that the current study only considered the recurrent interactions of two canonical cell types. Different classes of inhibitory interneurons exhibit diverse cellular and synaptic properties, microcircuit connectivity patterns, and neurophysiological responses. Another future direction is to incorporate distinct classes of inhibitory interneurons into circuit models, allowing for a more nuanced investigation of inhibitory dysfunction beyond the relatively coarse net effect of the E/I ratio. Overall, our findings from computational simulations help bridge a crucial gap between microcircuit dysfunctions and perturbed E/I balance at the macro scale in TLE.

The utility of a biomarker is often contingent upon its relationship with clinical measures and core symptoms. By leveraging the considerable range of disease duration in our patient cohort, we found significantly lower global as well as local Hurst exponent values in patients with long disease duration, more frequent electroclinical seizures, and a higher frequency of interictal epileptic spikes. This finding aligns with earlier cross‐sectional structural work on TLE with wider windows of disease duration or stages, which demonstrated greater cortical thinning in subgroups with long disease duration compared with those with short duration.^[^ ^70^, ^81^, ^108^, ^109^ ^]^ Together with prior evidence of cumulative metabolic alternations,^[^ ^41^, ^110^ ^]^ these findings indicate that TLE is likely a progressive neurological disorder. Our longitudinal analysis supports this view, showing progressive decreases in the Hurst exponent in TLE over the follow‐up period. Recurrent seizures and ongoing E/I dysfunction may contribute to epileptic discharges and secondary damage in other brain regions and, in turn, extend epileptogenic networks. The Hurst exponent, as a marker of E/I balance, not only reflects these dynamic pathological changes but also highlights potential future intervention targets. By monitoring the progressive change of this metric, clinicians could be able to identify brain regions that are susceptible to disease‐related compromise and target them therapeutically. Early intervention, especially in surgical candidates, could prevent adverse neural reorganization that exacerbates cognitive decline. In patients for whom surgery is delayed nevertheless, serial scanning may still enable the identification of potentially progressive effects, and targets for disease‐modifying therapies, ultimately improving individualized disease staging, intervention, and prognosis. In addition, the extent of Hurst exponent decreases correlated with deficits in high‐order cognitive functions in both our cross‐sectional and longitudinal studies. Previous research has linked structural and functional alterations in TLE to deficits in various cognitive domains (memory, language, and executive control).^[^ ^14^, ^42^, ^44^, ^50^ ^]^ For example, loss of volumes in prefrontal subregions has been associated with poor executive functioning^[^ ^111^ ^]^ and impaired memory.^[^ ^112^ ^]^ Decreased activity in frontoparietal regions has also been linked to poorer working memory.^[^ ^113^ ^]^ In our study, frontocentral and default mode areas were most affected, and the observed correlation with deficits in overall cognitive function (MoCA, EpiTrack) is plausible, considering the role of these large‐scale networks in complex cognitive tasks, such as working memory, reasoning, and language processing.^[^ ^114^, ^115^, ^116^ ^]^ A lower Hurst exponent value, therefore, reflects poorer cognitive performance, as dysregulated excitatory activity propagates and results in noisier, less efficient neural processing.^[^ ^117^ ^]^ We also found a positive relationship between progressive changes in the MoCA score and the Hurst exponent in TLE, further supporting the notion of cognitive decline over time.^[^ ^46^, ^118^ ^]^ Collectively, these findings suggest that Hurst exponent monitoring could index the progression of cortical E/I imbalance and cognitive impairment in patients with TLE. Future interventions, such as surgery or stimulation, could be guided by this metric, providing a novel way to tailor treatment to individual patients.

Altogether, our work revealed widespread imbalances of the cortical E/I ratio at the macroscale and explored their clinical, electrical, and cognitive implications. The present findings were replicated in two separate samples of intractable TLE and were consistent across a range of methodological parameters. However, several considerations should be taken into account when interpreting the findings. First, we opted to use the Hurst exponent of neural time series to estimate local E/I ratio alternations in TLE, which has been recently proposed to capture local changes in the synaptic E/I ratio. Multimodal datasets incorporating simultaneous measurements of metabolism (e.g., PET or MRS), pathology, and, neuroimaging factors may ultimately provide a more comprehensive assessment of TLE‐related E/I imbalances. Second, we identified potential disease epicenters using cross‐sectional data, precluding reconstruction of the temporal sequence of pathology. Modeling the spread of cortical E/I imbalance across brain regions over time remains an exciting open question that could eventually be addressed by large, multimodal longitudinal datasets in TLE.

## 4 Experimental Section

### Participants—Discovery Dataset (MICA‐MICs)

Forty individuals with pharmaco‐resistant TLE [17 males; mean ± SD age = 35.80 ± 11.04 years (18–63 years)] were studied, who underwent MRI examination for research purposes at the Montreal Neurological Institute and Hospital between 2018 and 2023. TLE diagnosis and lateralization of seizure focus as left (*n* = 27) and right TLE (*n* = 13) followed ILAE criteria,^[^ ^52^ ^]^ and were determined by a comprehensive evaluation that included detailed history, review of medical records, neuropsychological assessment, video‐EEG recordings, and clinical MRI. Based on qualitative MRI reading, 14 patients were considered to have marked hippocampal alterations compatible with hippocampal sclerosis ipsilateral to the seizure focus.^[^ ^119^, ^120^, ^121^ ^]^ This was complemented by quantitative analysis of MRI‐based hippocampal atrophy and/or asymmetry, as well as FLAIR images, using previously established method.^[^ ^122^ ^]^ They suggested hippocampal structural alterations in 24 patients. In the 15 patients who underwent neurosurgical operation at the time of study, histological analysis based on ILAE criteria confirmed hippocampal sclerosis or gliosis in 9. For further details, please see Table S1 (Supporting Information). The proportion was consistent with previous studies from the group,^[^ ^14^, ^39^, ^40^, ^123^ ^]^ and international multicentric assessments.^[^ ^124^ ^]^ The healthy control group included 40 adults with no history of neurological or psychiatric conditions [19 males; 34.25 ± 3.98 years (28–44 years)] who underwent MRI scans using the same imaging protocol as the TLE group.^[^ ^51^ ^]^ There were no differences in age (*t* = 0.84, *p* = 0.406) and sex (*χ* ^2^ = 0.20, *p* = 0.653) between TLE and healthy control groups. Detailed demographic and clinical information are provided in Table 1.

### Participants—Replication Dataset (EpiC)

This dataset consisted of 30 pharmaco‐resistant TLE patients [10 males; 30.87 ± 11.46 years (18–58 years)] who had undergone research‐dedicated MRI scans at Universidad Nacional Autónoma de Mexico between 2013 and 2017.^[^ ^45^, ^48^ ^]^ A similar evaluation classified patients as left (*n* = 18) or right TLE (*n* = 12). Quantitative analysis of T1‐weighted and FLAIR images revealed hippocampal sclerosis in 24 (80.00%) patients, in the form of volume loss and/or FLAIR hyperintense signals. Patients were compared to 30 healthy adults [11 males; 31.83 ± 11.35 years (18–57 years)] who had no history of neurological or psychiatric illness and underwent the same imaging protocol. As in the discovery dataset, there were no differences in age (*t* = 0.33, *p* = 0.744) and sex (*χ* ^2^ = 0.07, *p* = 0.787) between TLE and healthy control groups. Detailed demographic and clinical information are provided in Table 1.

### Patient Consent Statement

All studies were approved by local Ethics Committees (*MICA‐MICs*: Montreal Neurological Institute and Hospital, McGill University, project NO. 2018–3469; *EpiC*: Institute of Neurobiology, Universidad Nacional Autónoma de Mexico, project NO. 019. H‐RM). Written informed consent was obtained from all participants according to the declaration of Helsinki.

### MRI Acquisition—Discovery Dataset (MICA‐MICs)

All participants (healthy controls and patients) underwent baseline multimodal MRI scans, including T1‐weighted, diffusion‐weighted, and resting‐state fMRI. Fifteen patients underwent 1.81 ± 1.20 years of follow‐up scans, of which five additionally underwent 3.40 ± 0.55 years of follow‐up scans. All scans were acquired prior to surgery using a 3.0T Siemens Magnetom Prisma‐Fit scanner equipped with a 64‐channel head coil. Two T1‐weighted scans were acquired using a 3D MPRAGE sequence (TR = 2300 ms, TE = 3.14 ms, FA = 9°, FOV = 256 × 256 mm^2^, voxel size = 0.8 × 0.8 × 0.8 mm^3^, matrix size = 320 × 320, 224 slices). Diffusion‐weighted MRI data were acquired using a 2D EPI sequence (TR = 3500 ms, TE = 64.4 ms, FA = 90°, FOV = 224 × 224 mm^2^, voxel size = 1.6 × 1.6 × 1.6 mm^3^, 3 b0 images, b‐values = 300/700/2000 s/mm^2^ with 10/40/90 diffusion directions). Resting‐state fMRI data were acquired using a multiband accelerated 2D EPI sequence (TR = 600 ms, TE = 30 ms, FA = 52°, FOV = 240 × 240 mm^2^, voxel size = 3 × 3 × 3 mm^3^, matrix size = 80 × 80, multi‐band factor = 6, 48 slices, 700 volumes). A subset participant (28 controls and 27 patients) additionally underwent pseudo‐continuous arterial spin labeling (ASL) MRI (TR = 4150 ms, TE = 10 ms, FA = 90°, voxel size = 4.5 × 4.5 × 7 mm^3^, FOV = 288 × 288 mm^2^, post‐label delay = 1550 ms, 14 slices).

### MRI Acquisition—Replication dataset (EpiC)

All participants had multimodal MRI scans (T1‐weighted, diffusion MRI, and resting‐state fMRI), of which 14 TLE patients had follow‐up scans with a mean interscan interval of 2.61 ± 0.89 years (range = 0.7–4 years). All scans were acquired prior to surgery using a 3.0T Philips Achieva MR scanner, and included i) one T1‐weighted MRI scan (3D gradient‐echo EPI, TR = 8.1 ms, TE = 3.7 ms, FA = 8°, FOV = 256×256 mm^2^, voxel size = 1 × 1 × 1 mm^3^, 240 slices), ii) one resting‐state fMRI scan (2D gradient‐echo EPI, TR = 2000 ms, TE = 30 ms, FA = 90°, voxel size = 2 × 2 × 3 mm^3^, 34 slices, 200 volumes), and iii) one diffusion‐weighted MRI scan (2D EPI, TR = 11.86 s, TE = 64.3 ms, FOV = 256 × 256 mm^2^, voxel size = 2 × 2 × 2 mm^3^, 2 b0 images, b‐value = 2000 s mm^−2^, 60 diffusion directions).

### MRI Preprocessing

MRI data from the *MICA‐MICs* and *EpiC* datasets were processed using virtually identical pipelines via *micapipe* (version 0.2.2; http://micapipe.readthedocs.io),^[^ ^125^ ^]^ an open multimodal MRI pipeline that integrates AFNI, FSL, FreeSurfer, ANTs, MRtrix, and Workbench.^[^ ^126^, ^127^, ^128^, ^129^, ^130^ ^]^ T1‐weighted data underwent gradient non‐uniformity correction, re‐orientation, skull stripping, intensity normalization, and tissue segmentation. Diffusion‐weighted data underwent denoising, b0 intensity normalization, and correction for susceptibility distortion, head motion, and eddy current. Resting‐state fMRI processing involved discarding the first five volumes, re‐orientation, motion, and distortion correction. Nuisance variable signals were removed using an ICA‐FIX classifier.^[^ ^131^ ^]^ Volumetric time series were non‐linearly co‐registered to native FreeSurfer space with boundary‐based registration,^[^ ^132^ ^]^ and mapped to individual mid‐thickness surfaces with trilinear interpolation. Cortical time series were resampled to the Conte69 surface space (with ≈32k vertices/hemisphere) and smoothed with a 10‐mm full‐width half‐maximum (FWHM) kernel. ASL MRI data were processed using FSL‐BASIL (https://asl‐docs.readthedocs.io). The *oxford_asl*, an automated command line utility within BASIL, was used to generate a calibrated map of absolute resting‐state tissue perfusion for each participant. For further details, see Ref. [133] Resulting cortical blood flow (CBF) map was co‐registered to the native FreeSurfer space using boundary‐based registration,^[^ ^132^ ^]^ projected onto the Conte69 surface space, and smoothed using a 10‐mm FWHM kernel. Lastly, subject‐specific vertex‐wise resting‐state fMRI time series and CBF maps were parcellated into 360 cortical regions defined by the HCP multi‐modal parcellation (HCP‐MMP).^[^ ^134^ ^]^ The average time series for each subcortical structure was generated by averaging all voxels within that structure.

### Connectivity Matrix Generation

Functional connectivity (FC) was calculated as the correlation coefficient of the fully processed time series for each pair of regions (360×360). A functional connectivity dynamics (FCD) index was computed as follows. Each region's resting‐state fMRI time series (with a total length of 695‐time points) was segmented into 596 windows of 100 time points each (60 s), with an overlap of 99 time points. The whole‐brain FC matrix was constructed for each time window and vectorized by considering only the upper triangular entries. These vectorized matrices were then cross‐correlated, generating a 596 × 596 FCD matrix for each participant.

Individual structural connectivity (SC) was generated from preprocessed diffusion‐weighted data via MRtrix.^[^ ^130^ ^]^ Anatomically‐constrained tractography was first performed using tissue types (cortical and subcortical gray matter, white matter, cerebrospinal fluid) derived from each participant's processed T1‐weighted images registered to native DWI space.^[^ ^135^ ^]^ Multi‐shell and multi‐tissue response functions were estimated and constrained spherical deconvolution and intensity normalization were performed.^[^ ^136^, ^137^ ^]^ The tractogram was then generated based on a probabilistic approach with 40 million streamlines, a maximum tract length of 250 and a fractional anisotropy cut‐off of 0.06. Subsequently, spherical deconvolution‐informed filtering of tractograms (SIFT2)^[^ ^138^ ^]^ was applied to reconstruct the whole‐brain streamlines weighted by cross‐sectional multipliers. Lastly, the SC matrix (360×360) was constructed by mapping the reconstructed cross‐section streamlines onto the HCP‐MMP atlas with 360 nodes, in which the connection weights between nodes were defined as the weighted streamline count.

### Hurst Exponent Analysis

Prior work using in silico modeling and in vivo chemogenetic manipulations had validated the utility of the Hurst exponent, an index estimated from neural time‐series data,^[^ ^5^ ^]^ to infer the underlying alternations in the synaptic E/I ratio. In these reports, the Hurst exponent decreased as the E/I ratio shifted toward higher excitation. Here, the Hurst exponent of each brain region's preprocessed resting‐state fMRI time series was calculated and used as a proxy of the overall E/I ratio within that area. In brief, for each participant, each brain region's time series was modeled as fractionally integrated processes, and the corresponding Hurst exponent was estimated using the univariate maximum likelihood method and discrete wavelet transform.^[^ ^5^, ^139^ ^]^ The specific function utilized was the *bfn_mfin_ml.m* function from the *nonfractal* toolbox (https://github.com/wonsang/nonfractal),^[^ ^140^ ^]^ with the “filter” argument set to “haar” and the “ub” and “lb” arguments set to [1.5, 10] and [−0.5, 0], respectively.

To contextualize the regional pattern of the Hurst exponent to a range of molecular, structural, and functional features, relevant cortical maps were obtained from BigBrainWarp toolbox (https://bigbrainwarp.readthedocs.io)^[^ ^141^ ^]^ and neuromaps (https://netneurolab.github.io/neuromaps/).^[^ ^56^ ^]^ Specifically, the maps of the sensory‐fugal axis of cytoarchitectural differentiation,^[^ ^142^ ^]^ cortical thickness,^[^ ^71^ ^]^ intracortical myelination (T1w/T2w ratio),^[^ ^143^ ^]^ gene expression gradient,^[^ ^57^ ^]^ and neurotransmitter/receptor gradient were fetched and parcellated.^[^ ^58^, ^68^ ^]^ Spearman rank correlations separately quantified the spatial correlation between each brain annotation and the group‐level Hurst exponent map in healthy controls. Statistical significance (i.e., *P*_spin_) of correlation coefficients between cortical maps was assessed non‐parametrically via comparison against a null distribution of null maps with preserved spatial autocorrelation (spin tests) with 5000 iterations,^[^ ^66^ ^]^ implemented using ENIGMA Toolbox (https://enigma‐toolbox.readthedocs.io).^[^ ^144^ ^]^

### Statistical Analysis

Before statistical analysis, region‐wise Hurst exponent values in TLE patients were initially normalized relative to healthy controls, and sorted into ipsilateral/contralateral to the epileptogenic focus.^[^ ^145^ ^]^ Surface‐based linear models assessed group differences in each brain area's Hurst exponent between patients and healthy controls using BrainStat (https://brainstat.readthedocs.io).^[^ ^146^ ^]^ The effect size was calculated as Cohen's *d*. Age and sex were controlled, and findings were corrected for false discovery rate (FDR) using random field theory for nonisotropic images.^[^ ^147^, ^148^ ^]^ For regions surviving FDR correction, *post‐hoc* analyses using two‐sample *t*‐tests were conducted. To assess community‐wise group differences, the 360 nodes of the whole brain were first stratified according to their network assignments based on the Cole‐Anticevic Brain‐wide Network Partition (CAB‐NP)^[^ ^59^ ^]^ defined on the HCP‐MMP atlas (i.e., Glasser), Yeo‐7 functional networks,^[^ ^60^ ^]^ and von Economo classes.^[^ ^61^ ^]^ The mean Hurst exponent value was calculated for each network/class in each individual, and compared between groups using two‐sample *t*‐tests, with significance thresholded at *P*_FDR_ < 0.05. Finally, to demonstrate the robustness of the findings with respect to head motion, the mean framewise displacement from resting‐state fMRI scans was calculated for each participant. The surface‐wide comparison of the Hurst exponent was then repeated while additionally controlling for the mean framewise displacement.

### Recurrent Neural Circuit Modeling

A biophysically‐based mean‐field model was used to simulate coordinated neuronal activities across the whole brain based on long‐range anatomical connection and to infer microcircuit‐level parameters of neuronal populations at a regional level. Specifically, a parametric mean‐field model (pMFM)^[^ ^26^ ^]^ was harnessed that captures the link between time‐varying functional dynamics of intrinsic brain activity and structural connection, as well as its modulation through region‐specific microcircuit parameters. In comparison to other models that also incorporate local microcircuit properties,^[^ ^27^, ^63^ ^]^ the pMFM, by allowing them to vary along the anatomical and functional hierarchical axes of the cerebral cortex,^[^ ^26^, ^57^ ^]^ generate more realistic simulations of large‐scale brain dynamics with modest parametric complexity. A comprehensive description of pMFM, including the mathematical details of the mean‐field model, can be found in refs.[26, 63, 65] In brief, the pMFM assumes that the neural dynamics of a given brain region were governed by four components: i) recurrent (intra‐regional) input, where a larger recurrent input current corresponds to a stronger recurrent connection strength *w*; ii) inter‐regional input, mediated by structural connection strength between a pair of regions and scaled by a global scaling constant *G*; iii) external input *I*, mainly from subcortical structures; iv) neuronal noise, assumed to be Gaussian with a standard deviation *σ*. Here, recurrent connection strength *w*, external input current *I*, and noise amplitude *σ* varied across brain regions, while *G* was kept constant. Additionally, *w*, *I*, and *σ* were parameterized as linear combinations of group‐level T1w/T2w myelin maps^[^ ^143^ ^]^ and the principal gradient of resting‐state functional connectivity,^[^ ^149^ ^]^ rather than varying independently:

(1) $$w_{i}=a_{w}\mathrm{My}e_{i}+b_{w}\mathrm{Gra}d_{i}+c_{w}$$

(2) $$I_{i}=a_{I}\mathrm{My}e_{i}+b_{I}\mathrm{Gra}d_{i}+c_{I}$$

(3) $$\delta_{i}=a_{\delta }\mathrm{My}e_{i}+b_{\delta }\mathrm{Gra}d_{i}+c_{\delta }$$

where *w*_i_, *I*_i_, and *σ*_i_ were the recurrent connection strength, external input current, and noise amplitude of the *i*‐th cortical region, respectively. Mye_i_ and Grad_i_ denoted the average scores of T1w/T2w MRI estimates of intracortical myelin and the principal resting‐state FC gradient in the *i*‐th cortical region derived from HCP 100 unrelated healthy adults. Therefore, there were a total of 10 unknown parameters (*a*_w_, *b*_w_, *c*_w_, *a*_I_, *b*_I_, *c*_I_, *a*_σ_, *b*_σ_, *c*_σ_, *G*) to be estimated by maximizing fit to empirical static FC and FCD (Figure 2a).

In this study, for each group (healthy controls, or TLE), 40 participants were randomly subdivided into training (*n* = 15), validation (*n* = 15), and test (*n* = 10) sets. Group‐level SC and FC matrices (360 × 360) were computed by averaging the FC and SC matrices across participants separately within the training, validation, and test sets. FCD matrices could not be directly averaged across participants as there was no temporal correspondence of resting‐state fMRI time series between participants. The cumulative distribution function (CDF) of each participant's FCD matrix was constructed by collapsing the upper triangular entries, and then simply averaged across all participants separately within the training, validation, and test sets, which was referred to as a group FCD CDF.^[^ ^26^ ^]^ Subsequently, in the training set, the CMA‐ES algorithm^[^ ^64^ ^]^ was iterated 50 times and repeated 5 times with different random initializations, yielding 250 candidate parameter sets. The 250 candidate parameter sets were evaluated in the validation set. The top 10 candidate parameter sets selected from the validation set based on the model fit were tested in the test set to determine the optimal set of parameters (with the lowest cost). More specifically, the simulated fMRI signal from each parameter set was used to compute a 360 × 360 static FC matrix and a 596 × 596 FCD matrix. The agreement between the simulated and empirical static FC matrices was defined as Pearson's correlation (*r*) between the upper triangular entries of the two matrices, in which a larger *r* indicated a more similar static FC. The disagreement between the simulated and empirical FCD matrices was defined as the Kolmogorov–Smirnov (KS) distance^[^ ^150^ ^]^ between the two matrices’ CDF, in which a smaller KS distance indicated a more similar FCD. Following previous work, an overall cost was defined as [(1–*r*) + KS] to optimize both static FC and FCD^[^ ^26^ ^]^; lower cost thus implied a better fit to empirical static FC and FCD. For robustness, the split of participants into training, validation, and test sets was repeated five times for each group. Finally, the best parameter sets from the five splits were averaged, yielding the representative set of parameters for each group.

Spearman correlations were calculated between the group‐level pFMF parameters (Figure 2b) and Hurst exponent (Figure 1a) to evaluate the association between variations in regional Hurst exponent and microcircuit properties. Regional alternations in microcircuit parameters (*w* and *I*) between TLE and healthy controls were quantified by simply subtracting their group‐level parameter scores. These alternations were then correlated with the effect size of regional group differences in the Hurst exponent (i.e., Cohen's *d* in Figure 1b). Significances of spatial correlations were determined via spin permutation tests, with 5000 iterations.

### Network Spreading Mapping

Group‐average SC and FC matrices derived from an independent sample (i.e., HCP) of 100 unrelated healthy participants were used to estimate the mean alternation of neighbors of each brain region.^[^ ^71^ ^]^ Briefly, neighbors of a given brain region *i* were defined as regions connected to it with a structural connection, as defined by the SC matrix. The structurally connected neighbor alternation of node *i* (*D_i_*) was estimated as the average weighted alternation of all of *i*’s all neighbors,^[^ ^68^ ^]^ where *d*_j_ was the alternation (i.e., Cohen's *d*) of the *j*‐th neighbor of node *i*, *SC*_ij_ was the SC strength between node *i* and node *j*, and *N*_i_ was the total number of neighbors that were connected to node *i* (i.e., node degree).

(4) $$D_{j}=\frac{1}{N_{i}}\sum_{j=1}^{N_{i}}d_{j}\times SC_{ij},\;j\neq i$$

Structurally‐ and functionally‐defined neighbor alternation was estimated using the same equation as above, with the exception that regional alternation was additionally weighted by the FC strength to node *i* (*FC*_ij_)^[^ ^68^ ^]^:

(5) $$D_{j}=\frac{1}{N_{i}}\sum_{j=1}^{N_{i}}d_{j}\times SC_{ij}\times FC_{ij},\;j\neq i$$

Altogether, a single neighbor alternation value was estimated for each condition of each brain area. Spearman correlation coefficients were used to assess the relationship between node alternation and mean alternation of structurally‐defined neighbors, and both structurally‐ and functionally‐defined neighbors, separately. Spatial autocorrelation‐preserving spin tests were used to assess the statistical significance of correlations across brain regions. To ensure that the observed correlation was determined by the actual topology of the structural connection between brain regions rather than the basic spatial embedding of the connectome,^[^ ^151^ ^]^ a rewired null model was additionally used by generating surrogate networks that preserve the geometry of the structural connectome.^[^ ^66^, ^152^ ^]^ Specifically, the edges of the consensus network were first binned according to inter‐regional Euclidean distance. Edge pairs were then randomly swapped within each length bin. This procedure was repeated 1000 times, generating a population of rewired structural networks that preserve the nodal degree of the original network and that approximately preserve the edge length distribution of the empirical network. The *p*‐value (i.e., *P*_rewired_) was calculated as the fraction of correlations in null models that exceed the empirical correlation.

### Disease Epicenters Mapping

Disease epicenters were identified by spatially correlating each brain region's healthy structural and functional connectivity profiles (from the same HCP dataset) to the map of Hurst exponent alterations in TLE (i.e., un‐thresholded Cohen's *d* map in Figure 1b).^[^ ^70^ ^]^ This approach was repeated systematically across all brain regions with spin permutation tests at *P*_spin_ < 0.05. The higher the spatial similarity between a node's connectivity profile and the whole‐brain patterns of Hurst exponent disruption, the more likely this structure represented a disease epicenter (Figure 3b), regardless of its alternation level. Resulting likelihoods (i.e., correlation coefficients) were then ranked in descending order, with highly ranked brain regions representing disease epicenters. As for the hippocampus and subcortical regions, structural and functional connectivity profiles were systematically compared to whole‐brain patterns of Hurst exponent differences (i.e., Cohen's *d*) and assessed significance of correlations using spin permutation tests.

### Associations with Clinical and Cognitive Variables

For those regions showing significant between‐group differences (Figure 1b), the effect of disease severity (age at seizure onset, disease duration, and number of antiepileptic drugs) on the level of Hurst exponent alternations in TLE patients was assessed. The analyses were performed in the cross‐sectional and longitudinal cohorts combined (57 MRI scans in total). Linear mixed‐effect models were fitted containing *participant* intercept as a random term, and each clinical variable as a fixed term,^[^ ^40^ ^]^ and tested for a negative effect of the given clinical variable. Associations between the Hurst exponent and the number of electroclinical seizures captured during hospitalization were examined using Pearson's *r*. In addition, the effect of interictal epileptiform discharges (IEDs) in the temporal lobe on Hurst exponent alternations was explored in patients who underwent extended video‐EEG telemetry (mean ± SD = 8.68 ± 2.58 days, range = 2–15 days). For each TLE patient, the IEDs prevalence was obtained based on the classification from clinical EEG reports during hospitalization. Following the ACNS Critical Care EEG Terminology 2021,^[^ ^153^ ^]^ TLE patients were divided into two subgroups: rare IEDs (<1/h, *n* = 11) and prevalent IEDs (i.e., occasional/frequent/abundant, ≥1/h, *n* = 27). Differences in the Hurst exponent between the two subgroups were tested using two‐sample *t*‐tests.

To unveil the neurophysiological substrate of Hurst exponent alternations observed, further analysis on cortical blood flow (CBF) was conducted. CBF was tightly linked to brain metabolism,^[^ ^154^, ^155^ ^]^ varies across the lifespan,^[^ ^156^, ^157^ ^]^ and was increasingly recognized as a key neuroimaging biomarker for various neuropsychiatric and neurological disorders.^[^ ^154^, ^158^ ^]^ In this work, 28 healthy controls and 27 patients underwent ASL MRI. To compare regional CBF with corresponding Hurst exponent values, Each region's average CBF score and Hurst exponent in healthy controls was computed, then calculated Spearman's correlation between them; the *p*‐value was determined via spin permutation tests with 5000 iterations. Additionally, how well TLE‐related alternations in the Hurst exponent reflected CBF alternations were assessed. The global average CBF score and the average CBF score in brain regions showing significant Hurst exponent alternations (Figure 1b) were separately computed for each participant. Subsequently, CBF values were compared between TLE patients and healthy controls using two‐sample *t*‐tests and correlated CBF with subject‐specific Hurst exponent values while controlling for age and sex.

Neuropsychological assessments at the time of study MRI were also available for most participants, including general cognitive function [Montreal Cognitive Assessment (MoCA);^[^ ^73^ ^]^ 39 controls and 38 patients], and attention and executive functions (EpiTrack;^[^ ^74^ ^]^ 38 controls and 28 patients). TLE patients were directly compared to healthy controls using two‐sample *t*‐tests. To examine the clinical significance of cortical E/I imbalance, the Hurst exponent was separately extracted for the entire brain and in those significant regions, then correlated them with cognitive measurements described above, controlling for age and sex. In a separate analysis of TLE patients for whole longitudinal data was available, progressive alternations in Hurst exponent, MoCA, and EpiTrack scores were qualified by calculating the differences between baseline and follow‐up scans, and examining their associations.

### Case‐Control Classification

A supervised machine learning algorithm, implemented in LIBSVM,^[^ ^75^ ^]^ was utilized to assess the clinical utility of the Hurst exponent estimate in discriminating TLE patients from healthy controls. Classifier training and performance were evaluated using four‐fold cross‐validation with 1000 iterations. Features included 14 subcortical regions and the top 10%/20%/30%/40%/50% of cortical regions (i.e., 36/72/108/144/180 cortical regions, respectively) showing TLE‐control differences. Specifically, TLE patients were compared to healthy controls within the training set of each iteration, and then selected the top 10%/20%/30%/40%/50% of cortical regions to train classifiers. This ensured the same number of regions being selected in each training iteration while preventing data leakage. Performance was assessed using accuracy and area under the curve (AUC) of the receiver‐operating characteristic (ROC) curves. Statistical significance was determined using 1000 permutation tests with randomly shuffled participant labels.

### Replication Analysis

Hurst exponent alterations between TLE patients and healthy controls were validated in an independent dataset (i.e., *EpiC*; 30 healthy controls and 30 TLE) to verify the robustness of the findings. The protocols used for the analyses were the same as those described above. Briefly, differences in the average Hurst exponent across the entire brain and only within the significant regions identified in the discovery sample (see Figure 1b) were separately compared between TLE patients and healthy controls using two‐sample *t*‐tests. Surface‐wide differences in the Hurst exponent between TLE and healthy control groups were assessed via surface‐based linear models. The spatial correspondence between *MICA‐MICs* and *EpiC* datasets for the effect sizes (i.e., Cohen's *d*) of Hurst exponent alternations was examined via spin permutation tests with 5000 iterations. The effect of disease severity on Hurst exponent alternations across time was investigated. The average Hurst exponent value was separately calculated across the entire brain or within significant regions from the follow‐up scans, for each patient for whole longitudinal data was available (*n* = 14), then compared it to those derived from the baseline scans. The relationship between the Hurst exponent and epilepsy duration in TLE was accessed across both cross‐sectional and longitudinal scans using linear mixed‐effects models that contained *participant* intercept as a random term and *epilepsy duration* as a fixed term.

Finally, brain‐behavior associations were validated. At the *EpiC* site, a subset of participants (21 healthy controls and 28 TLE patients) underwent the Wechsler Memory Scale (WMS‐IV) test that consisted of seven subtests designed to assess memory performance.^[^ ^48^ ^]^ Every participant's performance was reported as five index scores: auditory memory (AMI), visual memory (VMI), visual working memory (VWMI), immediate memory (IMI), and delayed memory (DMI). These indices were normalized with respect to a Mexican population and adjusted for age and education level, and then were entered into a principal component analysis to reduce dimensionality. The loading score of the first principal component (i.e., PC1), accounting for 81% of the variance, was correlated with individual Hurst exponent values, with age and sex as covariates.

## Conflict of Interest

The authors declare no conflict of interest.

## Code Availability Statement

MRI processing was conducted using micapipe (http://micapipe.readthedocs.io).^[^ ^125^ ^]^ The Hurst exponent was computed using the nonfractal toolbox (https://github.com/wonsang/nonfractal). Surface‐based statistics were conducted using BrainStat (https://brainstat.readthedocs.io).^[^ ^146^ ^]^ Computational modeling was conducted using the parametric mean filed model (MFM) (https://github.com/HeavenBluer/Parametric‐MFM‐Project).^[^ ^26^ ^]^ Spin permutation tests of spatially cortical correlations were conducted using the ENIGMA Toolbox (https://enigma‐toolbox.readthedocs.io).^[^ ^144^ ^]^ Geometry‐preserving null networks for rewired tests were generated using https://www.brainnetworkslab.com/coderesources.^[^ ^152^ ^]^ The LIBSMV toolbox is available at https://www.csie.ntu.edu.tw/~cjlin/libsvmtools/.^[^ ^75^ ^]^

## Supporting information



Supporting Information

## Data Availability

The data that support the findings of this study are available from the corresponding author upon reasonable request.
