# Supplementary material for: Temporal Lobe Epilepsy Perturbs the Brain‐Wide Excitation‐Inhibition Balance: Associations with Microcircuit Organization, Clinical Parameters, and Cognitive Dysfunction
Source: Adv Sci (Weinh). 2025 Jan 13;12(9):2406835. doi: 10.1002/advs.202406835 (PMC11884548; doi:10.1002/advs.202406835)
Supplement: Supplementary file 1 — Supporting Information [file ADVS-12-2406835-s001.docx]

Supplementary Information

**Temporal Lobe Epilepsy Perturbs the Brain-Wide Excitation-Inhibition Balance: Associations with Microcircuit Organization, Clinical**

**Parameters, and Cognitive Dysfunction**

Ke Xie^1^, Jessica Royer^1^, Raul Rodriguez-Cruces^1^, Linda Horwood^1^, Alexander Ngo^1^,

Thaera Arafat^1^, Hans Auer^1^, Ella Sahlas^1^, Judy Chen^1^, Yigu Zhou^1^, Sofie L. Valk^2,3,4^,

Seok-Jun Hong^5,6,7^, Birgit Frauscher^8^, Raluca Pana^9^, Andrea Bernasconi^1^,

Neda Bernasconi^1^, Luis Concha^10^ & Boris C. Bernhardt^1^

**Author Affiliations:**

^1^ McConnell Brain Imaging Centre, Montreal Neurological Institute and Hospital, McGill University, Montreal, QC, Canada

^2^ Otto Hahn Research Group for Cognitive Neurogenetics, Max Planck Institute for Human Cognitive and Brain Sciences, Leipzig, Germany

^3^ Institute of Neurosciences and Medicine (INM-7), Research Centre Jülich, Jülich, Germany

^4^ Institute of Systems Neuroscience, Heinrich Heine University Düsseldorf, Düsseldorf, Germany

^5^ Center for Neuroscience Imaging Research, Institute for Basic Science, Sungkyunkwan University, Suwon, South Korea

^6^ Department of Biomedical Engineering, Sungkyunkwan University, Suwon, South Korea

^7^ Center for the Developing Brain, Child Mind Institute, New York City, NY, USA

^8^ Department of Neurology and Department of Biomedical Engineering, Duke University, Durham, NC, USA

^9^ Montreal Neurological Institute and Hospital, McGill University, Montreal, QC, Canada

^10^ Institute of Neurobiology, Universidad Nacional Autónoma de Mexico, Queretaro, Mexico

**Correspondence to**:

Boris C. Bernhardt, PhD

McConnell Brain Imaging Centre,

Montreal Neurological Institute and Hospital,

McGill University, Montreal, QC, Canada

E-mail: boris.bernhardt@mcgill.ca


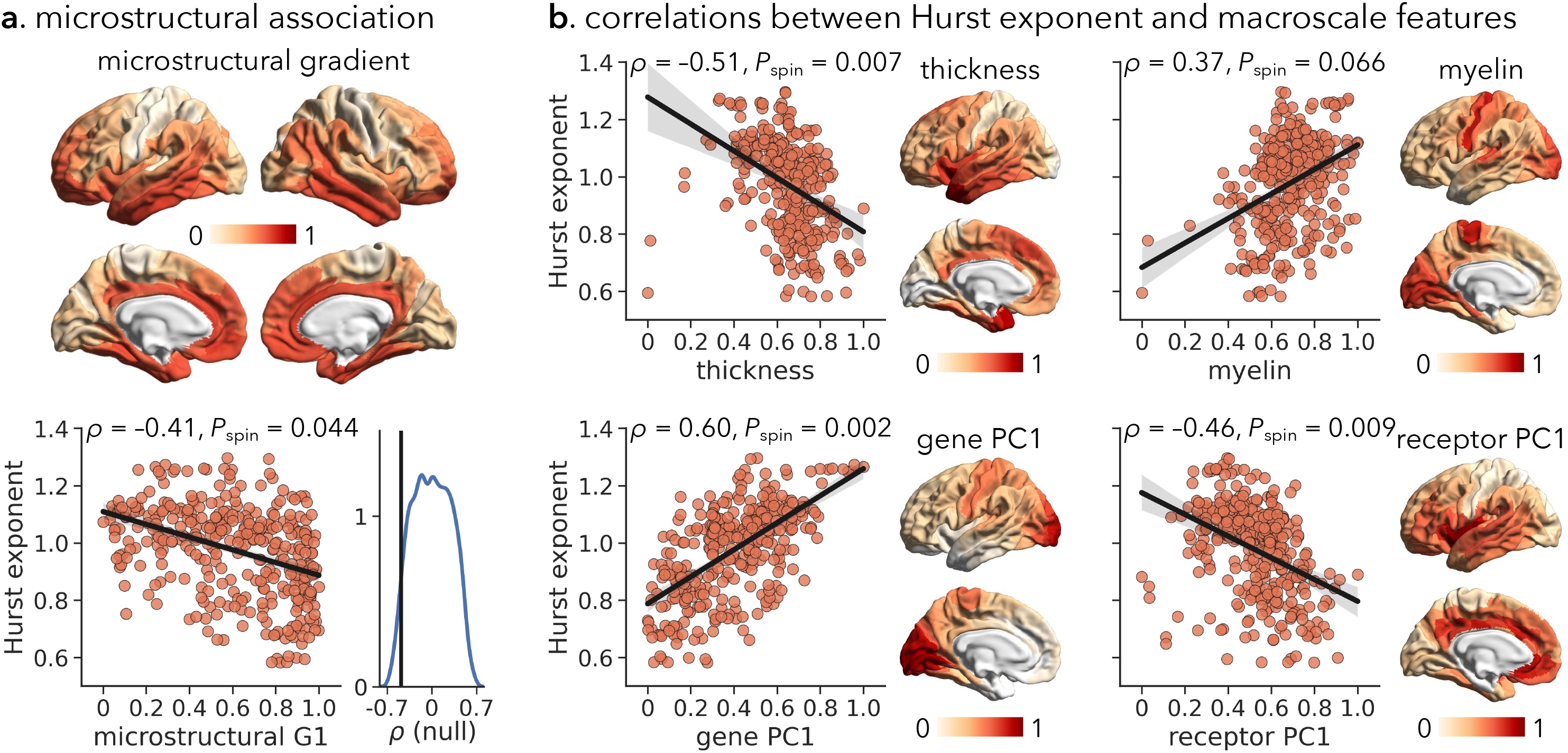


**Figure S1. Regional Hurst exponent values vary spatially across the brain**. **(a)** Across the neocortex, regional Hurst exponent values are negatively correlated with the sensory-fugal hierarchy of cytoarchitectural differentiation (“microstructural G1”). **(b)** Regional Hurst exponent value spatially aligns with the distribution of cortical thickness, intracortical myelination (T1w/T2w MRI ratio), the principal component of AHBA brain-specific gene expression data (“gene PC1”), and the principal component of neurotransmitter transporters/receptor density (“receptor PC1”). For example, lower Hurst exponent values (*i.e.*, greater E/I ratio) are observed in brain regions with lower intracortical myelination. The statistical significance of the spatial correlation between cortical brain maps (*i.e.*, *P*_spin_) is assessed non-parametrically using spin permutation tests (with 5,000 iterations) that control for spatial autocorrelation.


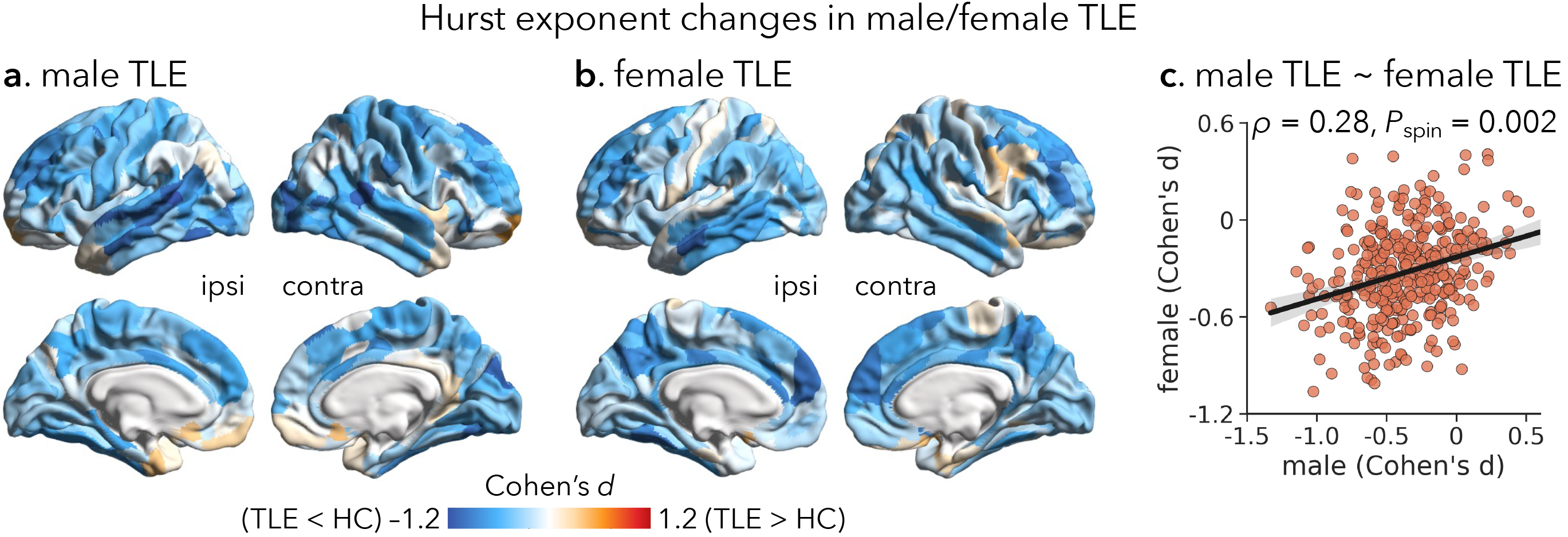


**Figure S2. Hurst exponent alterations in TLE vs controls in male/female participants**. **(a)** Regional Hurst exponent differences between male TLE patients and male controls. **(b)** Regional Hurst exponent differences between female TLE patients and female controls. **(c)** Spatial correlation of regional Hurst exponent abnormalities (*i.e.*, Cohen’s *d*) between male and female TLE patients. ipsi = ipsilateral; contra = contralateral.


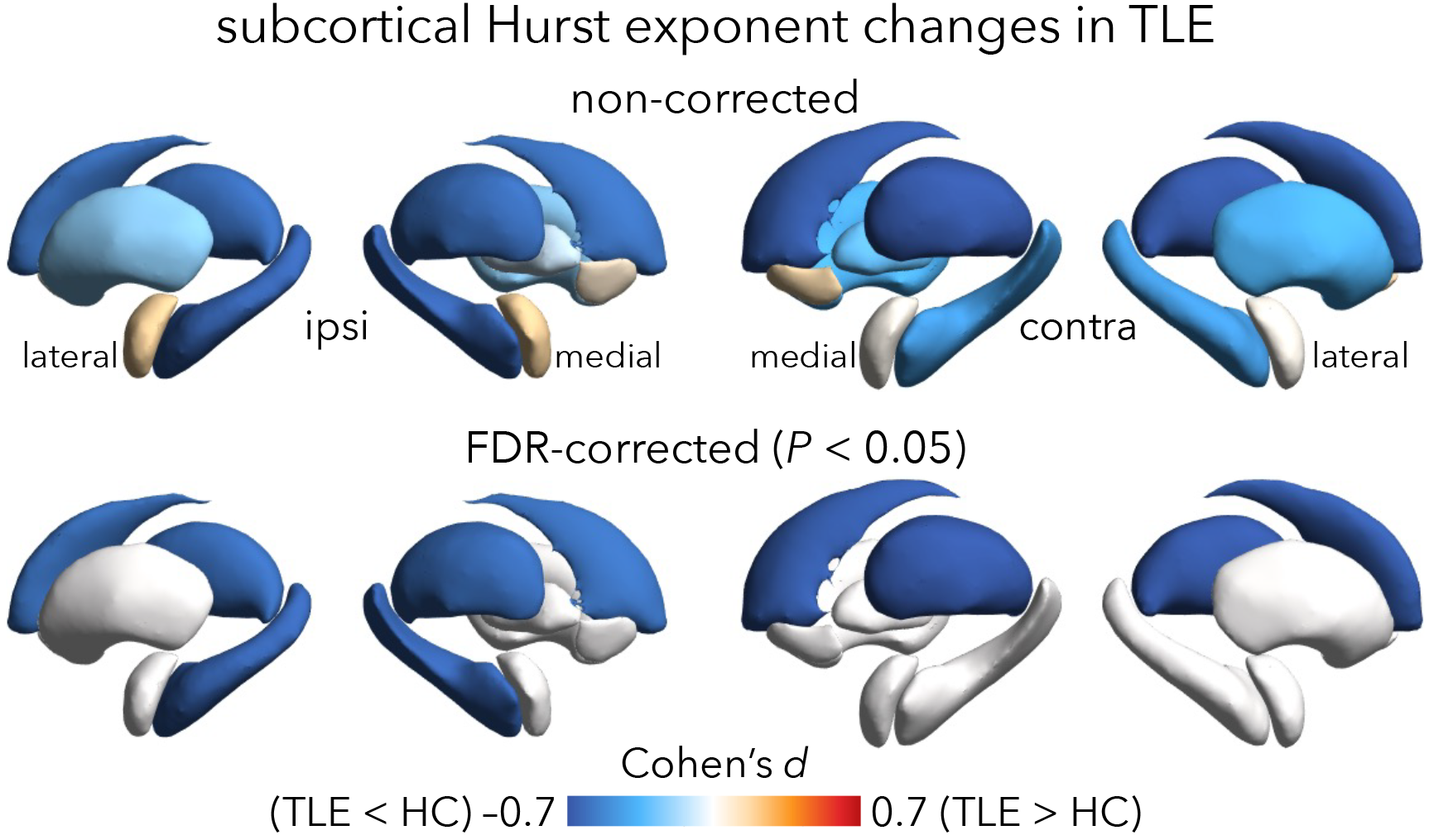


**Figure S3. Subcortical and hippocampal Hurst exponent alterations in TLE**. We found decreased Hurst exponent values in TLE patients compared to healthy controls in the ipsilateral hippocampus, as well as bilateral caudate and thalamus after correction for multiple comparisons (*P*_FDR_ < 0.05). ipsi = ipsilateral; contra = contralateral.


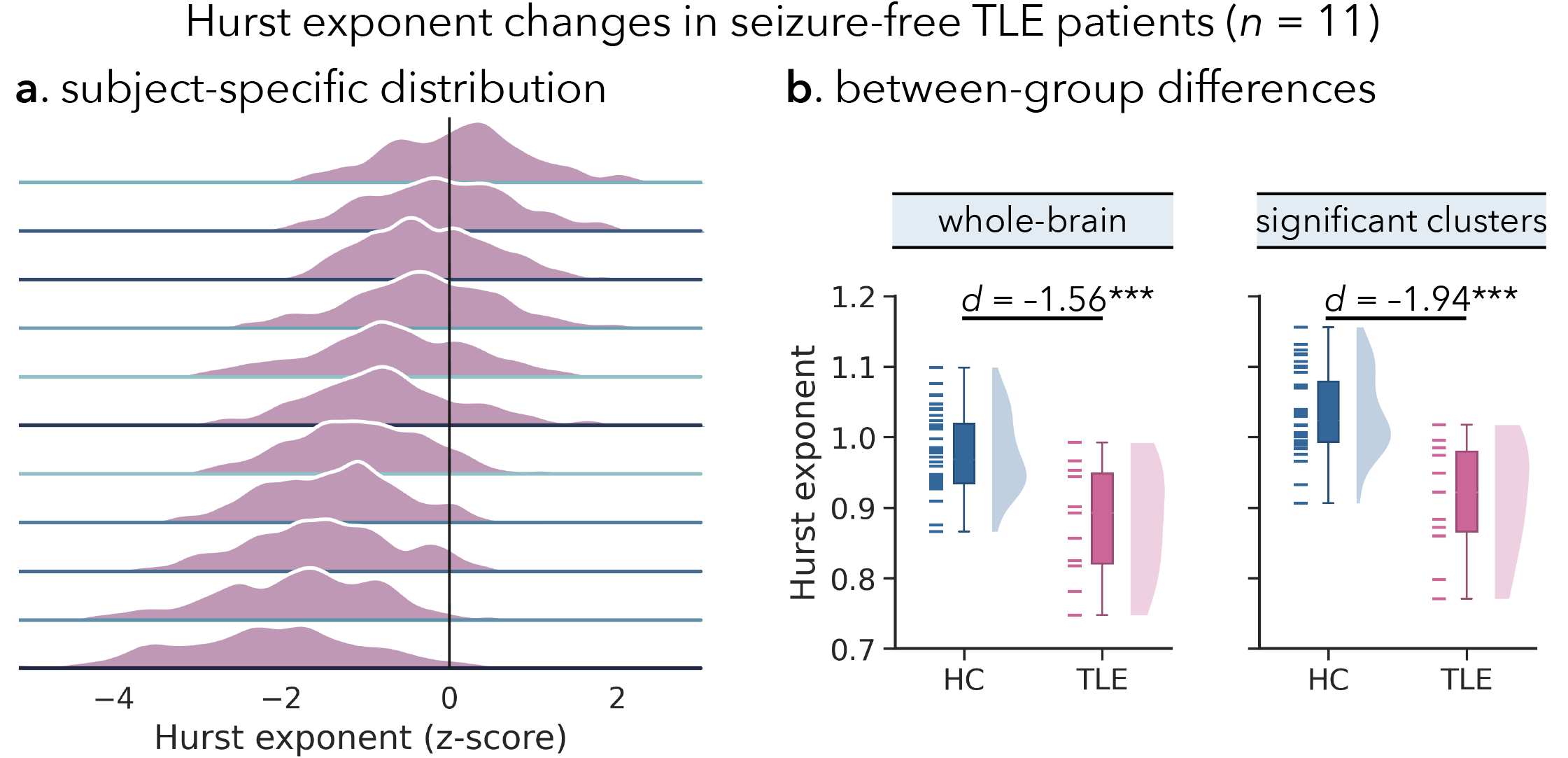


**Figure S4. Hurst exponent alterations in seizure-free TLE patients**. **(a)** Subject-specific distribution of regional Hurst exponent values (*z*-score relative to healthy controls) in seizure-free TLE patients (*n* = 11). **(b)** TLE-control differences in the average Hurst exponent values across the entire brain (left) or in significant brain regions (right) in **Figure 1b**. *** *P* < 0.001. HC = healthy control; TLE = temporal lobe epilepsy.


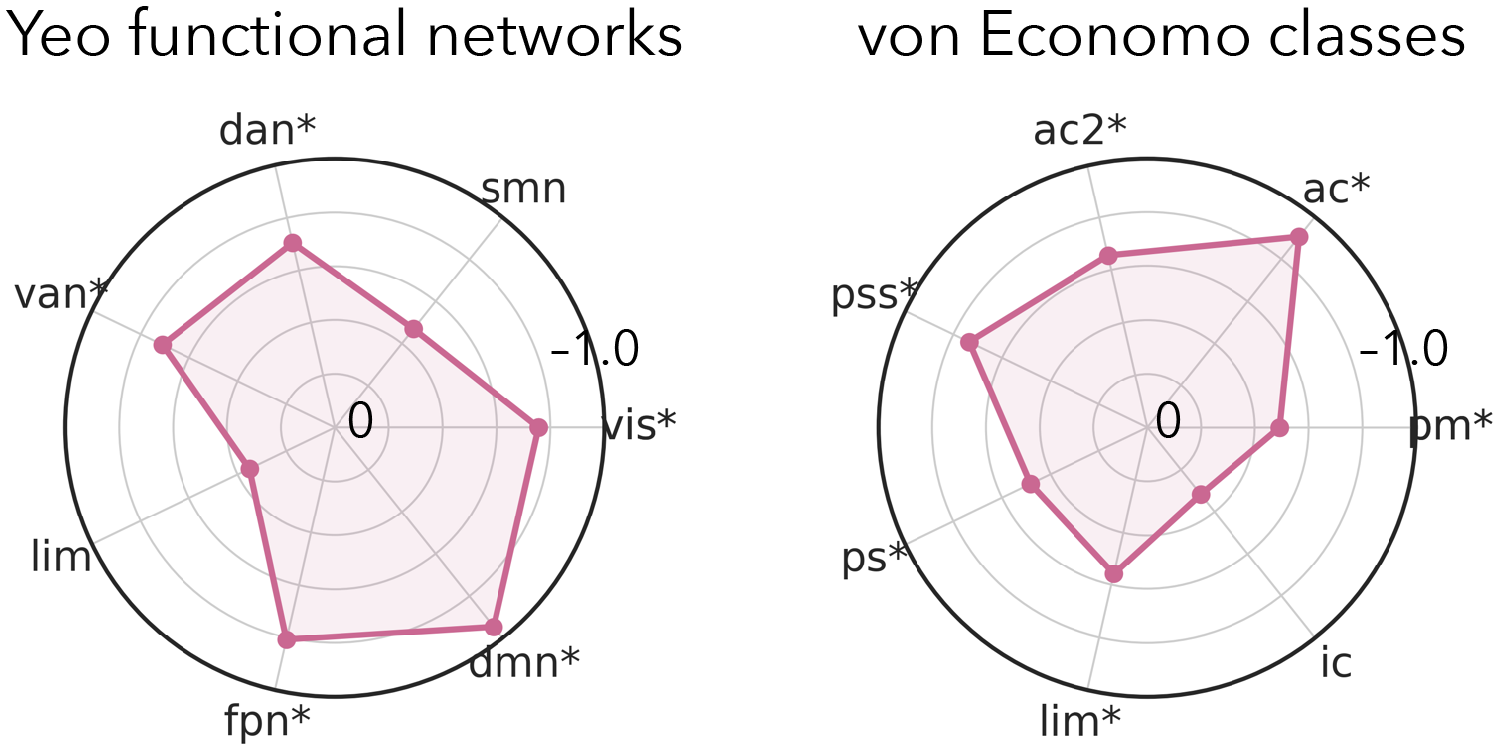


**Figure S5. Patterns of Hurst exponent alterations in intrinsic networks and cytoarchitectonic classes**. TLE-control differences (Cohen’s *d* values) in Hurst exponent values are calculated for Yeo 7 intrinsic functional networks (left) and von Economo cytoarchitectonic classes (right). More negative *d*-values indicate greater E/I ratio elevation in patients. * *P*_FDR_ < 0.05. Yeo-7 networks: vis = visual; smn = somatomotor; dan = dorsal attention; van = ventral attention; lim = limbic; fpn = frontoparietal; dmn = default mode. von cytoarchitectonic Economo classes: pm = primary motor cortex; ac/acc2 = association cortex; pss = primary/secondary sensory cortex; ps = primary sensory cortex; lim = limbic cortex; ic = insular cortex.


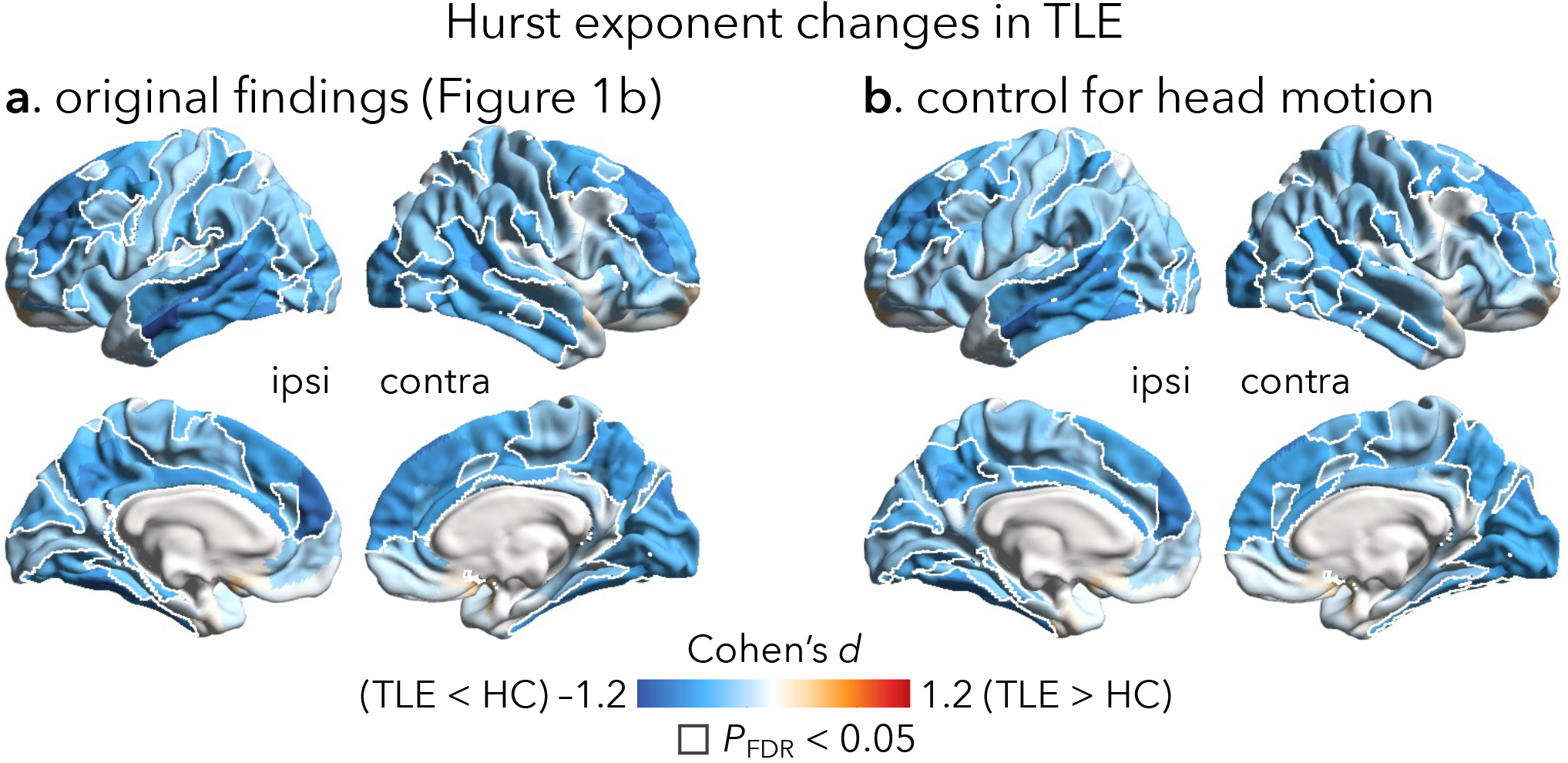


**Figure S6. Head motion effects**. Hurst exponent differences between TLE patients and healthy controls are shown without (**a**) and with (**b**) controlling for head motion. Surface-based findings are corrected for multiple comparisons using a false discovery rate (FDR) threshold of 0.05 (*P*_FDR_ < 0.05; white outlines). ipsi = ipsilateral; contra = contralateral.


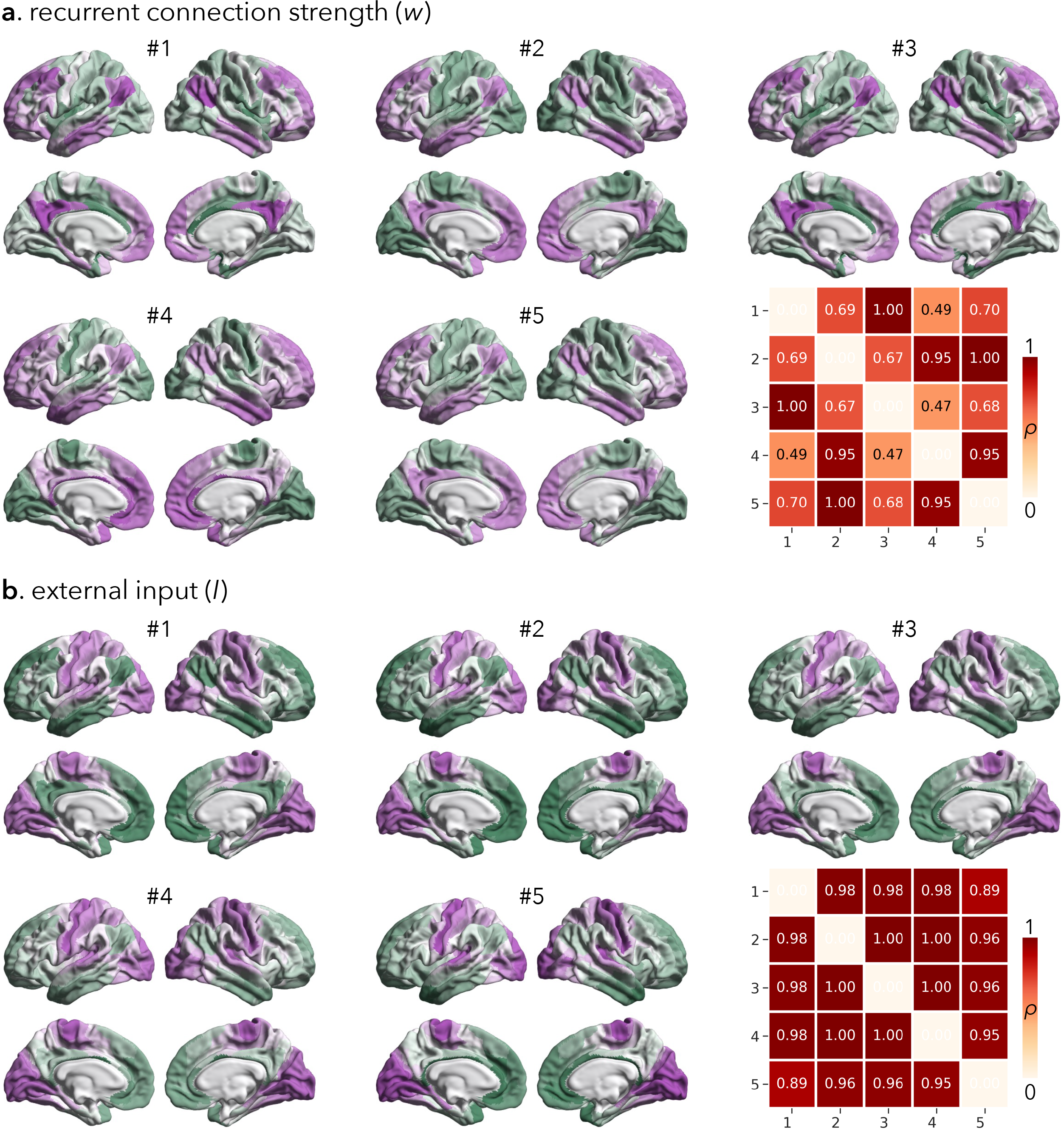


**Figure S7.** Cortex-wise recurrent connection strength (a) and external input current (b) of each iteration. Heat map matrix: spatial correlation coefficients between 5 iterations.


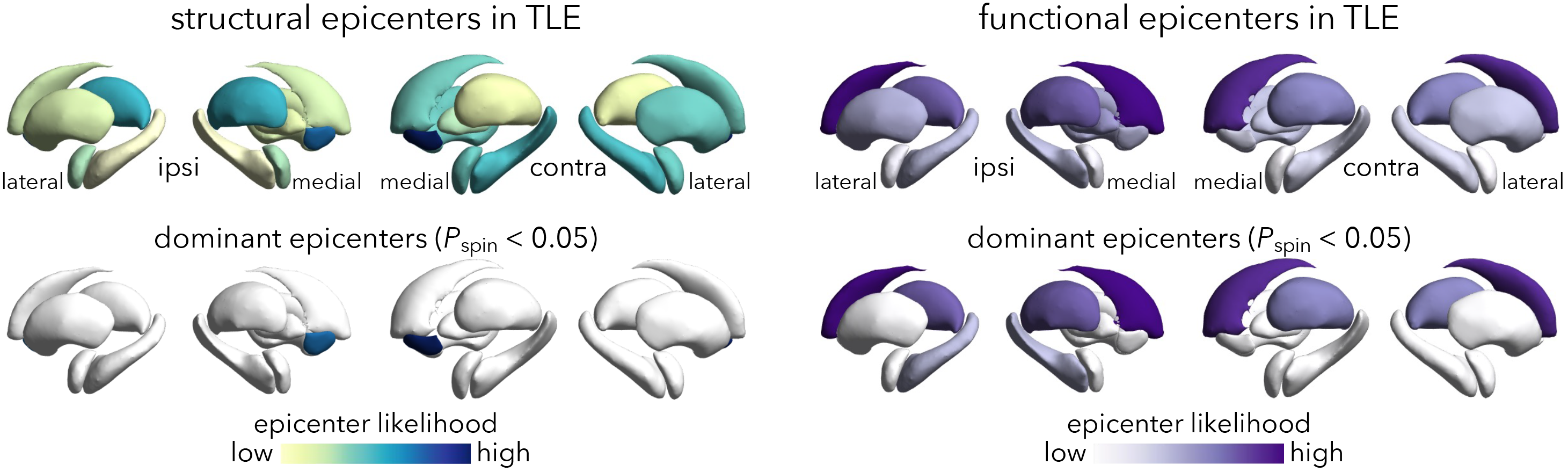


**Figure S8. Subcortical and hippocampal disease epicenters in TLE**. Epicenters are regions whose connectivity profiles significantly correlate with the spatial map of TLE-related Hurst exponent alterations; statistical significance is assessed using spin permutation tests. The highest-ranked structural (left) and functional (right) epicenters are in bilateral nucleus accumbens (*P*_spin_ < 0.05), and bilateral caudate and thalamus (*P*_spin_ < 0.05) and ipsilateral hippocampus (*P*_spin_ = 0.063), respectively. ipsi = ipsilateral; contra = contralateral.


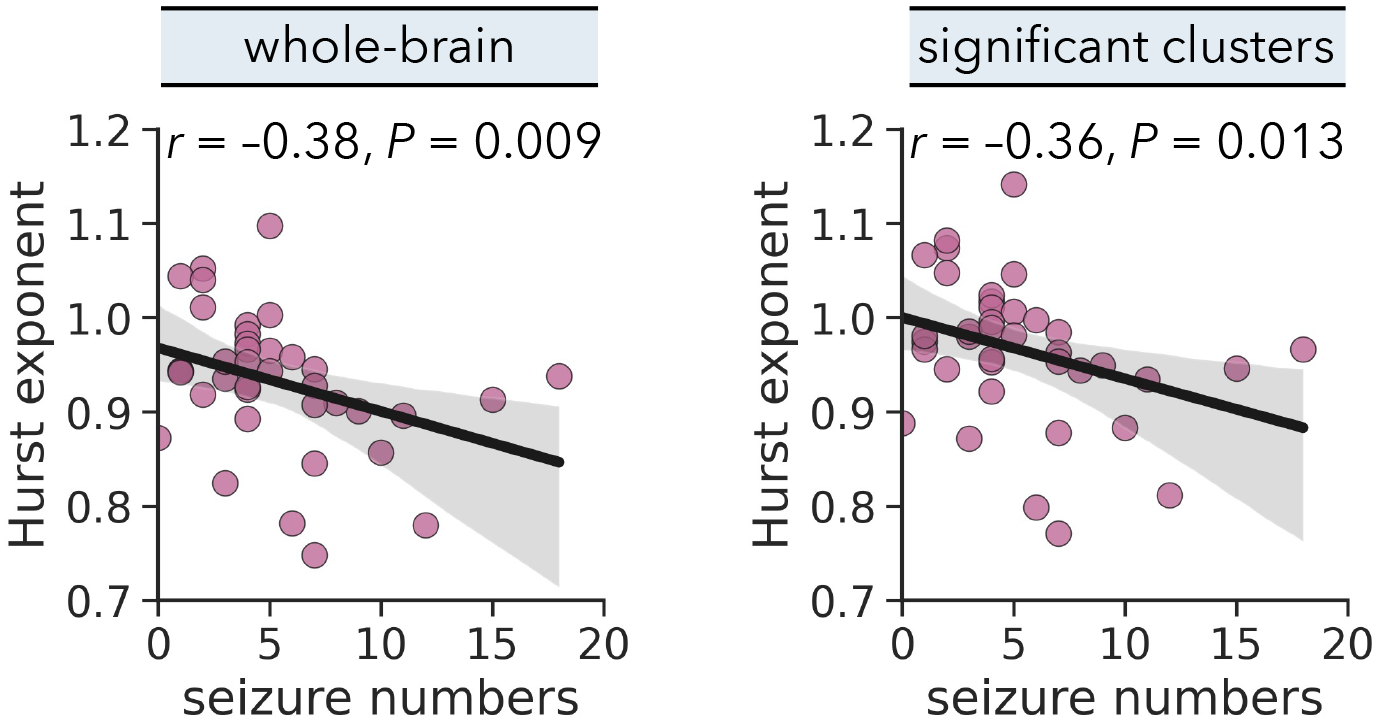


**Figure S9**. Correlations between the mean Hurst exponent and the number of electroclinical seizures captured during the EMU admission in TLE patients. Participant-specific average Hurst exponent value is calculated by averaging the Hurst exponent values across the entire brain (left) or in significant brain regions (right) in **Figure 1b**.


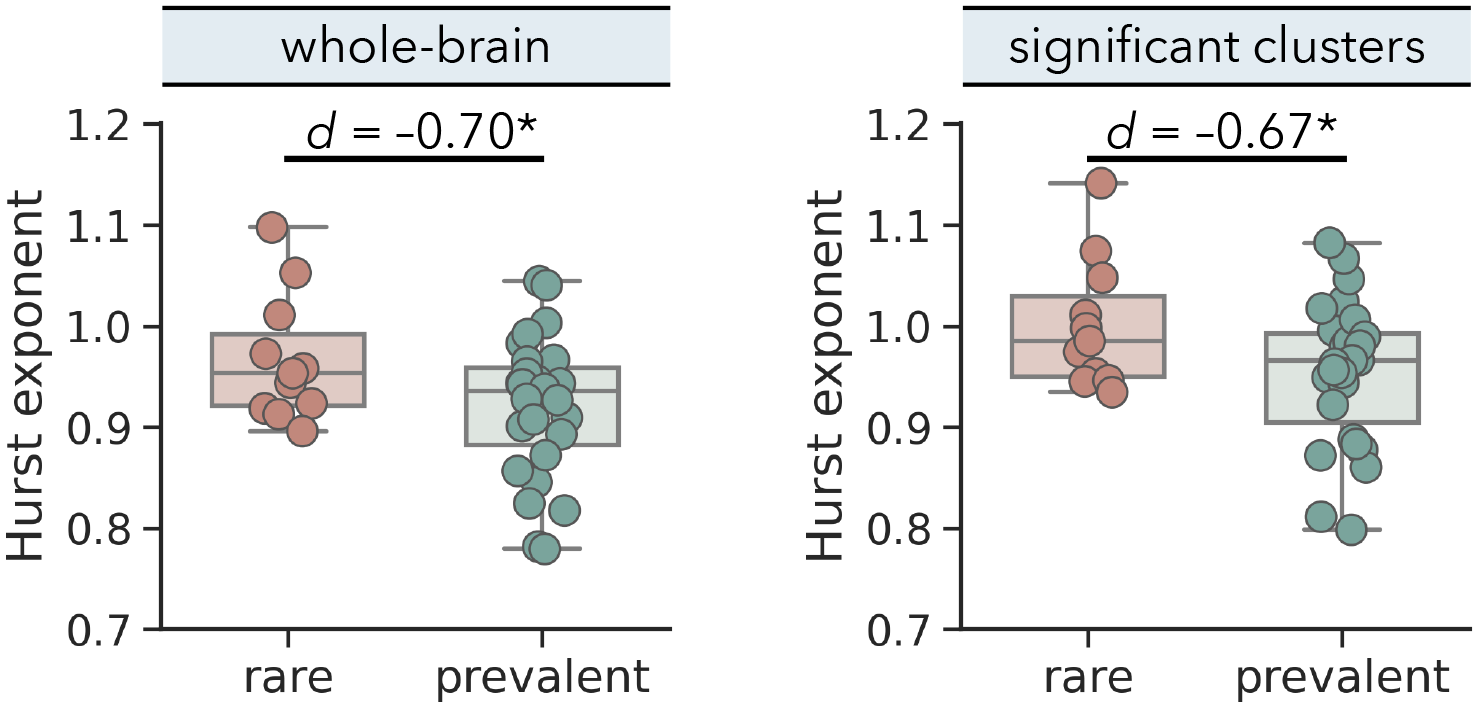


**Figure S10**. **Interictal epileptiform discharges (IEDs) effects**. Differences in the average Hurst exponent between patients with rare (*n* = 11) and prevalent (*i.e.*, occasional/frequent/abundant, *n* = 27) interictal epileptic discharges (IEDs). Participant-specific average Hurst exponent value is calculated by averaging the Hurst exponent values across the entire brain (left) or in significant brain regions (right) in **Figure 1b**.


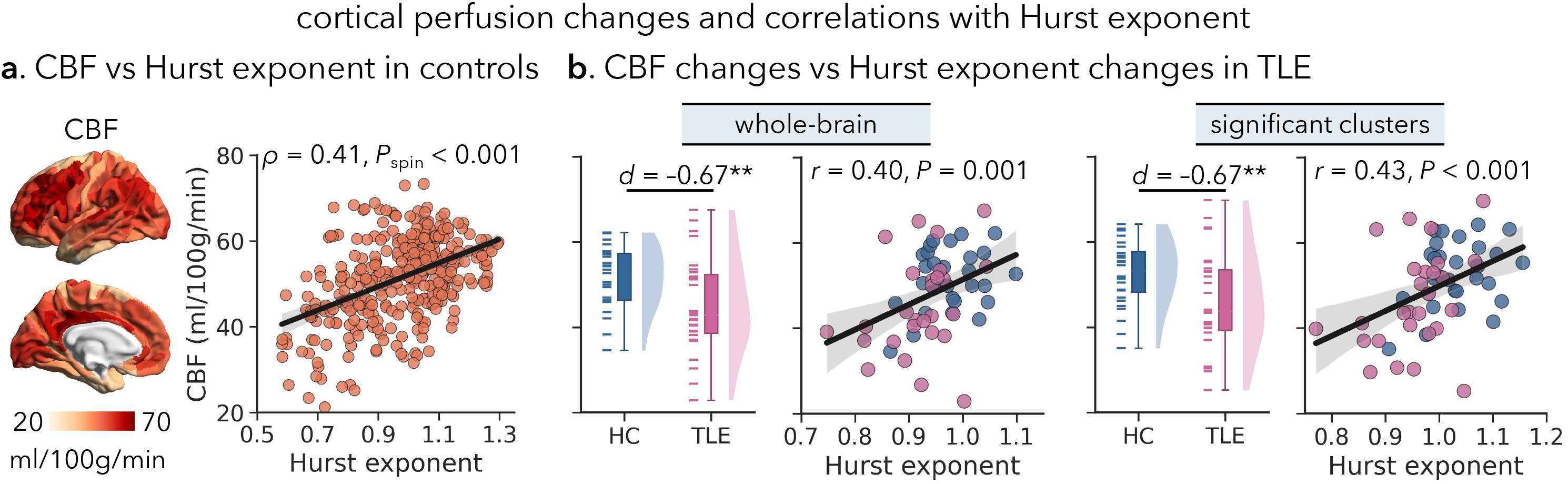


**Figure S11. Regional brain perfusion alterations in TLE and associations with Hurst exponent alterations**. **(a)** Regional Hurst exponent values spatially align with the spatial distribution of regional cortical blood flow (CBF) derived from resting-state arterial spin labeling (ASL) MRI. Brain regions with lower Hurst exponent (*i.e*., greater E/I ratio) tend to have lower perfusion. The statistical significance of the spatial correlation between brain maps (*i.e.*, *P*_spin_) is assessed non-parametrically via spin permutation tests with 5,000 iterations that preserved spatial autocorrelation. **(b)** Error bar plots: TLE-control differences in the mean CBF scores across the entire brain (left) or in brain regions (right) showing significant Hurst exponent differences (**Figure 1b**). Scatter plots: Associations between participant-specific mean Hurst exponent and CBF. ** *P* < 0.010. HC = healthy control; TLE = temporal lobe epilepsy.

**Table S1**. Patient-specific clinical information in the discovery site (MICA-MICs).

| **#** | **HS on qualitative**  **MRI reading** | **alteration on**  **quantitative MRI** | **HS on histopathology** |
| --- | --- | --- | --- |
| 01 | no | yes | – |
| 02 | no | yes | – |
| 03 | no | yes | – |
| 04 | no | yes | – |
| 05 | no | yes | no |
| 06 | no | no | yes |
| 07 | yes | yes | yes |
| 08 | yes | yes | yes |
| 09 | yes | no | – |
| 10 | no | no | – |
| 11 | yes | yes | – |
| 12 | yes | yes | yes |
| 13 | yes | yes | yes |
| 14 | no | no | – |
| 15 | no | no | no |
| 16 | yes | yes | – |
| 17 | no | yes | – |
| 18 | no | no | – |
| 19 | yes | yes | – |
| 20 | no | yes | no |
| 21 | no | no | – |
| 22 | no | no | – |
| 23 | no | no | – |
| 24 | no | yes | no |
| 25 | yes | yes | yes |
| 26 | yes | yes | yes |
| 27 | yes | yes | – |
| 28 | no | no | – |
| 29 | no | no | – |
| 30 | yes | yes | – |
| 31 | yes | yes | yes |
| 32 | yes | yes | yes |
| 33 | no | no | – |
| 34 | no | no | – |
| 35 | no | no | – |
| 36 | no | yes | no |
| 37 | no | yes | no |
| 38 | no | no | – |
| 39 | no | yes | – |
| 40 | no | no | – |

**Table S2**. Patient-specific antiseizure medications (ASMs) in the discovery site (MICA-MICs).

| **#** | **# ASMs** | **ASMs name** | **#** | **# ASMs** | **ASMs name** |
| --- | --- | --- | --- | --- | --- |
| 01 | 2 | LEV, CBZ | 21 | 4 | LCM, ESL, BRV, CZP |
| 02 | 3 | LEV, VPA, PB | 22 | 2 | LEV, CBZ |
| 03 | 3 | LTG, LCM, PER | 23 | 1 | CBZ |
| 04 | 1 | LEV | 24 | 3 | LTG, CZP, CBZ |
| 05 | 2 | LEV, LCM | 25 | 2 | ESL, LTG |
| 06 | 2 | CLB, LCM | 26 | 5 | LEV, RFM, CBZ, PER, PB |
| 07 | 3 | ESL, LTG, LEV | 27 | 1 | LEV |
| 08 | 2 | LEV, LCM | 28 | 3 | LEV, PB, CBZ |
| 09 | 1 | LTG | 29 | 2 | LTG, CBZ |
| 10 | 2 | LEV, CBZ | 30 | 2 | CLB, PHT |
| 11 | 1 | LCM | 31 | 3 | LCM, CLB, CBZ |
| 12 | 2 | LTG, PHT | 32 | 2 | LEV, LTG |
| 13 | 2 | LEV, CLB | 33 | - | - |
| 14 | 3 | LEV, LTG, VPA | 34 | 1 | LTG |
| 15 | 2 | LTG, PHT | 35 | 2 | LEV, LTG |
| 16 | 2 | CBZ, LZP | 36 | 2 | CLB, BRV |
| 17 | 3 | LEV, LCM, LTG | 37 | 4 | BRV, LCM, CBZ, TPM |
| 18 | 2 | CBZ, PB | 38 | 1 | LCM |
| 19 | 3 | LEV, LTG, PGB | 39 | 3 | LTG, LEV, LCM |
| 20 | 2 | CLB, VPA | 40 | 3 | LEV, LCM, CLB |

BRV = Brivaracetam; CBZ = Carbamazepine; CLB = Clobazam; CZP = Clonazepam; ESL = Eslicarbazepine Acetate; LCM = Lacosamide; LEV = Levetiracetam; LTG = Lamotrigine; LZP = Lorazepam; PB = Phenobarbital; PER = Perampanel; PHT = Phenytoin; PGB = Pregabalin; RFM = Rufinamide; TPM = Topiramate; VPA = Valproate.

ASMs with more cognitive side effects: CBZ, CZP, LZP, PB, PER, PHT, PGB, TPM, VPA; ASMs with fewer/no cognitive side effects: BRV, CLB, ESL, LCM, LEV, LTG, RFM
